# Supplementary material for: Percutaneous vertebroplasty versus percutaneous kyphoplasty for osteoporotic vertebral compression fractures: an umbrella review protocol of systematic reviews and meta-analyses
Source: BMJ Open. 2024 Feb 20;14(2):e075225. doi: 10.1136/bmjopen-2023-075225 (PMC10882401; doi:10.1136/bmjopen-2023-075225)
Supplement: Supplementary data [file bmjopen-2023-075225supp003.pdf]

AMSTAR 2 contains 16 items, and 7 of them are critical domains (items 2, 4, 7, 9, 11, 13, 15). For more details refer to Table.

Items	Content
2	protocol registered before the beginning of the review
4	adequacy of the literature search
7	justification for excluding individual studies
9	risk of bias (ROB) from individual studies included in the review
11	appropriateness of meta-analytical methods
13	consideration of ROB when interpreting the results of the review
15	assessment of presence and likely impact of publication bias
