# Supplementary material for: Percutaneous vertebroplasty versus percutaneous kyphoplasty for osteoporotic vertebral compression fractures: an umbrella review protocol of systematic reviews and meta-analyses
Source: BMJ Open. 2024 Feb 20;14(2):e075225. doi: 10.1136/bmjopen-2023-075225 (PMC10882401; doi:10.1136/bmjopen-2023-075225)
Supplement: Author's manuscript [file bmjopen-2023-075225.draft_revisions.pdf]

BMJ Open is committed to open peer review. As part of this commitment we make the peer review history of every article we publish publicly available.

When an article is published we post the peer reviewers' comments and the authors' responses online. We also post the versions of the paper that were used during peer review. These are the versions that the peer review comments apply to.

The versions of the paper that follow are the versions that were submitted during the peer review process. They are not the versions of record or the final published versions. They should not be cited or distributed as the published version of this manuscript.

BMJ Open is an open access journal and the full, final, typeset and author-corrected version of record of the manuscript is available on our site with no access controls, subscription charges or pay-per-view fees (http://bmjopen.bmj.com).

If you have any questions on BMJ Open's open peer review process please email info.bmjopen@bmj.com

BMJ Open

Percutaneous Vertebroplasty vs Percutaneous Kyphoplasty for Osteoporotic Vertebral Compression Fractures: An Umbrella Review Protocol of Systematic Reviews and Meta-Analysis

Journal:	*BMJ Open*
Manuscript ID	bmjopen-2023-075225
Article Type:	Protocol
Date Submitted by the Author:	30-Apr-2023

Complete List of Authors:	Gao, Qingyang; Sichuan University West China Hospital, Department of Plastic and Burn Surgery<br>Li, Qiujiang; Sichuan University West China Hospital, Department of Orthopedics Surgery, Orthopedic Research Institute<br>Yang, Huiliang; Sichuan University West China Hospital, Department of Orthopedics, Orthopedic Research Institute,<br>Cen, Ying; Sichuan University West China Hospital, Department of Plastic and Burn Surgery

Keywords:	Aging, ORTHOPAEDIC & TRAUMA SURGERY, Bone diseases < ORTHOPAEDIC & TRAUMA SURGERY, Spine < ORTHOPAEDIC & TRAUMA SURGERY

SCHOLARONE™
Manuscripts

Percutaneous Vertebroplasty vs Percutaneous Kyphoplasty for Osteoporotic Vertebral Compression Fractures: An Umbrella Review Protocol of Systematic Reviews and Meta-Analysis

Qingyang Gao[a,┼], Qiujiang Li[b,┼], Huiliang Yang[b,*], Ying Cen[a,*]

a. Department of Plastic and Burn Surgery, West China Hospital, Sichuan University, Chengdu, 610041, P.R. China

b. Department of Orthopedics, Orthopedic Research Institute, West China Hospital, Sichuan University, Chengdu, 610041, P.R. China

* Correspondence:

Huiliang Yang, MD, Department of Orthopedics, Orthopedic Research Institute, West China Hospital, Sichuan University, 610041, P.R. China; Email: huiliang_yang@wchscu.cn

Ying Cen, MD, Department of Plastic and Burn Surgery, West China Hospital, Sichuan University, 610041, P.R. China; Email: cenying0104@163.com

┼ Qingyang Gao and Qiujiang Li contributed equally to this manuscript

For peer review only

Percutaneous Vertebroplasty vs Percutaneous Kyphoplasty for Osteoporotic Vertebral Compression Fractures: An Umbrella Review Protocol of Systematic Reviews and Meta-Analysis

Abstract

Introduction:

Numerous systematic reviews and meta-analyses have confirmed the safety and beneficial efficacy of percutaneous vertebroplasty and percutaneous kyphoplasty for patients with osteoporotic vertebral compression fractures. However, the variation among results is extensive, which hinders evaluation and use by clinicians. This study aims to investigate the efficacy and safety of these procedures in treating osteoporotic vertebral compression fractures, providing a more reliable evidence base for clinical practice.

Methods and analysis: We will search five databases (PubMed, Scopus, EMBASE, Cochrane Library and Web of Science) from inception to 31st March, 2023 for English-publish systematic review and meta-analysis studies comparing the overall safety and efficacy of percutaneous vertebroplasty and percutaneous kyphoplasty in patients with osteoporotic vertebral compression fractures. Three reviewers will screen citation titles and abstracts, and evaluate the full-text of each relevant record based on prespecified eligibility criteria. Any discrepancies in decisions between reviewers will be resolved through discussion. We assessed the methodological quality of the included studies according to A MeaSurement Tool to Assess systematic Reviews 2 checklist.

Results: The results of this study will be published in a peer-reviewed journal.

Ethics and dissemination: This umbrella review will provide valuable insights for clinical and policy decisions regarding the benefits and harms of percutaneous vertebroplasty versus percutaneous kyphoplasty for osteoporotic vertebral compression fractures. No primary data will be collected and individual patient information and endangering participant rights, thus ethics approval is not required. Findings will be reported through a peer-reviewed publication, conference presentations and the popular press.

Protocol registration number: PROSPERO CRD42021268141 (https://www.crd.york.ac.uk/PROSPERO/#joinuppage)

For peer review only

Strengths and limitations of this study

1.This will be a comprehensive review assessing the reliability of current evidence on percutaneous vertebroplasty in the management of osteoporotic vertebral compression fractures compared with percutaneous kyphoplasty.

2.This protocol was designed following Joanna Briggs Institute's guidelines for umbrella reviews and reported following the Preferred Reporting Items for Systematic Reviews and Meta-Analyses statement.

3.The results will affect clinical and policy decisions regarding the benefits and harms of percutaneous vertebroplasty for treating osteoporotic vertebral compression fractures.

4.The conclusion of this study might be challenged because of the poor quality and heterogeneity of the reviewed studies.

Abbreviations: OVCFs = osteoporotic vertebral compression fractures, PVP = percutaneous vertebroplasty, PKP = percutaneous kyphoplasty, PMMA = polymethylmethacrylate, RCT = randomized clinical trial, SRs/MAs = systematic reviews and meta-analysis, VAS = visual analog scale, ODI = Oswestry Disability Index, SF-36 = Short Form-36, CI = confidence intervals

Keywords: Osteoporotic Fractures, Compression Fractures, Vertebroplasty, Kyphoplasty

Introduction

As the society ages, osteoporotic vertebral compression fractures (OVCFs) caused by osteoporosis has become a major world-wide health issue [1, 2]. OVCFs often occur as a result of low-impact injuries and patients may not have a clear history of trauma. They primarily present with persistent back pain and local vertebral kyphosis, which significantly affects the survival rates and living standards of the elderly population [3-6].

Non-surgical therapies for OVCFs include bed rest and drug therapy [7, 8]. However, patients often receive poor pain relief from these methods and remain limitations in activities of daily living. Moreover, complications such as pressure sores, pneumonia, deep vein thrombosis, and urinary tract infections may arise during the treatment. Surgery treatments are usually applied to more serious fractures. Currently, percutaneous vertebroplasty (PVP) and percutaneous kyphoplasty (PKP)

For peer review only

have been widely used in OVCFs treatment. By injecting polymethylmethacrylate (PMMA) bone cement through a minimally invasive wound, PVP and PKP can provide immediate pain relief and stabilize the fractured vertebral body [9-13].

Nevertheless, the controversy over which method, PKP or PVP, is better for treating OVCFs persists. In a meta-analysis of six RCT studies involving 1077 patients, Zhu et al. Zhu et al. [14] performed a meta-analysis of six randomized clinical trial (RCT) studies involving a total of 1077 patients, and finally found that both PKP and PVP could improve function and relieve the pain, but PKP had lower cement leaking rates. By contrast, a meta-analysis of 16 studies aiming to evaluate the efficacy and safety of PKP/PVP for the treatment of OVCFs founded that PKP could better reduce bone cement leakage, restore vertebral height restoration and correct vertebral angulation in comparison to PVP [15]. The same conclusion is drawn by Chang et al. [16].

Systematic reviews and meta-analysis (SRs/MAs) are important research methods in evidence-based medicine and the best sources of clinical evidence [17] to create clinical guidelines and develop recommendations for medical practice. Although there are multiple published SRs/MAs studies on PKP/PVP for OVCFs, they produced varying and sometimes conflicting results, which could impact clinical decision-making.

To provide a comprehensive overview of these SRs/MAs studies, an umbrella review (or Systematic Review of Systematic Reviews) is needed. As another important part of evidence-based medicine, an umbrella review assesses the methodological and report quality of included SRs/MAs and provides the quality of evidence. This will be the first study to summarize systematic reviews and meta-analysis on clinical outcomes of PVP and PKP for the treatment of OVCFs. an umbrella review presented in our protocol will identify, collect, evaluate and integrate the existing knowledge of PKP/PVP for OVCFs to determine the more beneficial surgical treatments between PKP and PVP for patients with OVCFs and guide optimal treatment strategies. The results of this study will provide evidence for clinicians and further guide researchers to embark on relevant studies.

Methods and analysis

Study registration

The study protocol was registered on International prospective register of systematic reviews (PROSPERO) (https://www.crd.york.ac.uk/prospero) with a unique ID of CRD42021268141. It was designed using the methodology guidelines for umbrella reviews provided by the Joanna Briggs Institute (JBI) [18]. Meanwhile, this protocol will follow the guidance of the Preferred Reporting Items for Systematic

For peer review only

Reviews and Meta-analysis Protocols (PRISMA-P) [19] **(see online supplementary appendix 1 for the checklist).**

The research questions of this umbrella review were:

1) What is the efficacy (Primary outcomes) of PCP (Intervention) compared to PVP (Comparison) in OVCFs patients (Population)?

2) What is the safety (Secondary outcomes) of PCP (Intervention) compared to PVP (Comparison) in OVCFs patients (Population)?

3) To identify the pre-, peri-operative factors (Intervention and Comparison) that affect the functional improvements and radiographic improvements (Outcome) in OVCFs patients (Population)?

Patient and public involvement

No patients or the public will be involved in this study.

Search strategy

An electronic search will be conducted among various database, including PubMed、Scopus、EMBASE、Cochrane Library and Web of Science with the Medical Subject Headings (MeSH) and keywords “percutaneous vertebroplasty”, “percutaneous kyphoplasty”, “ balloon kyphoplasty”, “osteoporosis vertebral compression fractures”, “systematic reviews”, “meta-analysis”. The terms will be put into combinations with Boolean search syntax operators to retrieve the maximum number of articles.

As a supplement, the “related article” function from PubMed will be used to further identify potential articles that were eligible for inclusion in the review. In addition, grey literature and Google scholar was also searched. To include as many relevant studies as possible, we consulted with content experts. A draft search strategy for PubMed is provided in **Table 1.**

Eligibility criteria

Type of studies

We will include systematic reviews and meta-analysis studies comparing different clinical outcomes following PKP/PVP.

The studies should report the outcome data separately for the PKP and PVP groups.

Publication time

For peer review only

The dates range are set from database inception to 31st March, 2023.

Language of literature

The language of publication is restricted to English.

Inclusion criteria

We set the inclusion criteria for this umbrella review according to the population, intervention, comparison, outcome (PICO) principle [20].

Population:

Patients with vertebral compression fractures (VCFs) due to osteoporosis will be included. We impose a lower age limit >= 60 years to identify the elderly. Expect that, there was no restriction on gender, race or healthy status.

Intervention:

Interventions include PVP with curative intent

Comparison:

PKP with curative intent is considered as comparison

The volume of injected cement, fluoroscopy times, intraoperative blood loss and operation time were recorded for both intervention and comparison groups.

Outcomes

Primary outcomes include:

1) Functional outcomes : the visual analog scale (VAS) score , Oswestry Disability Index (ODI), Short Form-36 (SF-36);

2) Radiographic outcomes included Cobb angle and anterior vertebral body height.

Secondary outcomes include:

1) Bone cement leakage;

2) New vertebral compression fractures after the treatment

All outcomes will be assessed based on the definitions applied in the selected meta-analysis. Studies will not be included or excluded on the basis of reported outcomes.

Data collection

For peer review only

Study selection

First, literatures that do not meet the eligibility criteria will be excluded by screening the titles and abstracts. Then, the full-text of preliminary selected articles will be read by two reviewers (QY G and QJ L) independently to confirm the enrolled items according to the inclusion and exclusion criteria. Using a data extraction form, two reviewers(HR W and XC J) will extract the data, which is subsequently cross-checked by the first two reviewers (QY G and QJ L). If there is a disagreement in the checked results, arbitration will be resolved through discussion with a third reviewer (HL Y). The flowchart of literature selection process is shown in **Figure 1.**

Data extraction

Two reviewers (HR W and XC J) will extract data independently based on a tool developed by the Joanna Briggs Institute (JBI) Data Extraction form. When data extraction fails to reach an agreement, arbitration will be resolved through discussion with a third reviewer (HL Y). If data were depicted in graphs or incomplete, we will try to contact authors for obtaining original data by email.

Extracted data include:

1) basic information of the literature: first author, publication year range, country;

2) information of the study: study design included, sample size, search strategy，quality evaluation method， original research involved and interest disclosure;

3) population: gender, age, race, pain and deformity before treatment;

4) intervention/comparison: treatment, level of vertebra, the volume of injected cement, fluoroscopy times, intraoperative blood loss and operation time;

5) outcomes: primary outcome and secondary outcome as described above, time-point to obtain the outcomes;

6) conclusion.

For meta-analysis, we will extract summary effect sizes (random effect size and/or fixed effect size, 95% confidence intervals (CI)) and significance levels. When available, information of between-study heterogeneity (Cochrane Q statistic or I^2) and small-study effects will also be extracted.

Data synthesis and statistical analysis

A descriptive method including tables and graphs was done to describe the characteristics of the study participants was used to summarize study findings. Guidance from the Cochrane Handbook will be followed [21]. We will not report

For peer review only

study results as meta-analytically pooled effect estimates, due to multiple meta-analysis that may include information from overlapping individual studies. We will present a narrative synthesis of interventions and outcomes, and report the pooled treatment effect obtained from the highest quality and most comprehensive systematic reviews . The methodological quality of meta-analysis will be evaluated using the A Measurement Tool to Assess Systematic Reviews 2(AMSTAR 2) tool [22]. The relationship and findings between studies will be explored according to the narrative synthesis. In the final phase of the integrative review, we will describe and summarize key findings and conclusions of studies. Subgroup analysis considering the influence of sex, age, and outcomes will be conducted.

Identifying possible publication bias

We evaluate the reporting bias by funnel plot. To avoid the effect of subjective visual observation on funnel plot, Begg's and Egger's tests will be carried out to assess the asymmetry of funnel plot, as a supplementary approach.

Methodological quality evaluation

Two reviewers independently assess the assess the methodological quality of systematic reviews based on AMSTAR 2 [22]. When quality assessment results fail to reach an agreement, arbitration will be resolved through discussion with a third reviewer. The AMSTAR 2 is a reliable, valid and critical appraisal tool for systematic reviews that was applied to randomized and non-randomized studies of health care interventions[23, 24] **(see online supplementary appendix 2 for the checklist).** AMSTAR 2 contains 16 items, and 7 of them are critical domains (items 2, 4, 7, 9, 11, 13, 15). For more details refer to online supplementary appendix 3 [25]. The results of evidence quality for systematic reviews will be divided into four levels according to AMSTAR 2: critically low, low, medium, high.

Ethics and dissemination

There is no ethical approval or consent required for this systematic review study. The results of our study will be published on an international peer-reviewed journal and disseminated to the public through publications, conferences, and meetings.

Authors' contributions

All authors made substantive intellectual contributions in this study to qualify as

For peer review only

authors. QY G and QJ L developed the research question. QY G and QJ L registered the protocol and wrote the first draft of the manuscript. QY G and QJ L developed the search strategy and created the data extraction form. QY G and QJ L provided critical review and feedback at each stage of the process. All authors were involved in writing the manuscript. All authors read and approved the final manuscript.

Funding statement

This study was funded by The Science and Technology Project of the Health Planning Committee of Sichuan (No. 21PJ036) and National Natural Science Foundation of China (No. 82102521).

Competing interest's statement

The author declared no Competing interest's statement.

Patient consent for publication

Not applicable.

References:

1. Jin YZ, Lee JH, Xu B, Cho M: Effect of medications on prevention of secondary osteoporotic vertebral compression fracture, non-vertebral fracture, and discontinuation due to adverse events: a meta-analysis of randomized controlled trials. BMC Musculoskelet Disord 2019, 20(1):399.

2. Li HM, Zhang RJ, Gao H, Jia CY, Zhang JX, Dong FL, Shen CL: New vertebral fractures after osteoporotic vertebral compression fracture between balloon kyphoplasty and nonsurgical treatment PRISMA. Medicine (Baltimore) 2018, 97(40):e12666.

3. Bae IS, Kim JM, Cheong JH, Han MH, Ryu JI: Association between cerebral atrophy and osteoporotic vertebral compression fractures. PLOS ONE 2019, 14(11):e224439.

4. Wu XG, Zhang DY, Zhu BQ, Li AM: Efficacy of zoledronic acid with percutaneous kyphoplasty/vertebroplasty in the treatment of osteoporotic vertebral compression fractures: a systematic review and meta-analysis. Eur Rev Med Pharmacol Sci 2020, 24(23):12358-12367.

For peer review only

5. Zhu RS, Kan SL, Ning GZ, Chen LX, Cao ZG, Jiang ZH, Zhang XL, Hu W: Which is the best treatment of osteoporotic vertebral compression fractures: balloon kyphoplasty, percutaneous vertebroplasty, or non-surgical treatment? A Bayesian network meta-analysis. Osteoporos Int 2019, 30(2):287-298.

6. Yang S, Chen C, Wang H, Wu Z, Liu L: A systematic review of unilateral versus bilateral percutaneous vertebroplasty/percutaneous kyphoplasty for osteoporotic vertebral compression fractures. Acta Orthop Traumatol Turc 2017, 51(4):290-297.

7. Pan M, Ge J, Li Q, Li S, Mao H, Meng B, Yang H: Percutaneous vertebral augmentation in special Genant IV osteoporotic vertebral compression fractures. J Orthop Translat 2020, 20:94-99.

8. Long Y, Yi W, Yang D: Advances in Vertebral Augmentation Systems for Osteoporotic Vertebral Compression Fractures. PAIN RES MANAG 2020, 2020:3947368.

9. Zuo XH, Zhu XP, Bao HG, Xu CJ, Chen H, Gao XZ, Zhang QX: Network meta-analysis of percutaneous vertebroplasty, percutaneous kyphoplasty, nerve block, and conservative treatment for nonsurgery options of acute/subacute and chronic osteoporotic vertebral compression fractures (OVCFs) in short-term and long-term effects. Medicine (Baltimore) 2018, 97(29):e11544.

10. Li YX, Guo DQ, Zhang SC, Liang, Yuan K, Mo GY, Li DX, Guo HZ, Tang Y, Luo PJ: Risk factor analysis for re-collapse of cemented vertebrae after percutaneous vertebroplasty (PVP) or percutaneous kyphoplasty (PKP). INT ORTHOP 2018, 42(9):2131-2139.

11. Zhang H, Xu C, Zhang T, Gao Z, Zhang T: Does Percutaneous Vertebroplasty or Balloon Kyphoplasty for Osteoporotic Vertebral Compression Fractures Increase the Incidence of New Vertebral Fractures? A Meta-Analysis. PAIN PHYSICIAN 2017, 20(1):E13-E28.

12. Xiao H, Yang J, Feng X, Chen P, Li Y, Huang C, Liang Y, Chen H: Comparing complications of vertebroplasty and kyphoplasty for treating osteoporotic vertebral compression fractures: a meta-analysis of the randomized and non-randomized controlled studies. Eur J Orthop Surg Traumatol 2015, 25 Suppl 1:S77-S85.

13. Wang H, Sribastav SS, Ye F, Yang C, Wang J, Liu H, Zheng Z: Comparison of Percutaneous Vertebroplasty and Balloon Kyphoplasty for the Treatment of Single Level Vertebral Compression Fractures: A Meta-analysis of the Literature. PAIN PHYSICIAN 2015, 18(3):209-222.

14. Zhu Y, Cheng J, Yin J, Zhang Z, Liu C, Hao D: Therapeutic effect of

For peer review only

kyphoplasty and balloon vertebroplasty on osteoporotic vertebral compression fracture: A systematic review and meta-analysis of randomized controlled trials. Medicine (Baltimore) 2019, 98(45):e17810.

15. Wang B, Zhao CP, Song LX, Zhu L: Balloon kyphoplasty versus percutaneous vertebroplasty for osteoporotic vertebral compression fracture: a meta-analysis and systematic review. J ORTHOP SURG RES 2018, 13(1):264.

16. Chang X, Lv YF, Chen B, Li HY, Han XB, Yang K, Zhang W, Zhou Y, Li CQ: Vertebroplasty versus kyphoplasty in osteoporotic vertebral compression fracture: a meta-analysis of prospective comparative studies. INT ORTHOP 2015, 39(3):491-500.

17. Shrestha BM: Systematic Reviews and Meta-analysis: Principles and Practice. JNMA J Nepal Med Assoc 2019, 57(215):1-2.

18. Munn Z: Software to support the systematic review process: the Joanna Briggs Institute System for the Unified Management, Assessment and Review of Information (JBI-SUMARI). JBI Database System Rev Implement Rep 2016, 14(10):1.

19. Shamseer L, Moher D, Clarke M, Ghersi D, Liberati A, Petticrew M, Shekelle P, Stewart L, PRISMA-P Group. Preferred reporting items for systematic review and meta-analysis protocols (PRISMA-P) 2015: elaboration and explanation. BMJ. 2015 Jan 2;349(jan02 1):g7647.

20. Richardson WS, Wilson MC, Nishikawa J, Hayward RS: The well-built clinical question: a key to evidence-based decisions. ACP J Club 1995, 123(3):A12-A13.

21. Cumpston M, Li T, Page MJ, Chandler J, Welch VA, Higgins JP, Thomas J: Updated guidance for trusted systematic reviews: a new edition of the Cochrane Handbook for Systematic Reviews of Interventions. Cochrane Database Syst Rev 2019, 10:D142.

22. Kim HR, Choi CH, Jo E: A Methodological Quality Assessment of Meta-Analysis Studies in Dance Therapy Using AMSTAR and AMSTAR 2. Healthcare (Basel) 2020, 8(4).

23. Franco J, Meza N: Authors should also report the support for judgment when applying AMSTAR 2. J CLIN EPIDEMIOL 2021.

24. Pieper D, Puljak L, Gonzalez-Lorenzo M, Minozzi S: Minor differences were found between AMSTAR 2 and ROBIS in the assessment of systematic reviews including both randomized and nonrandomized studies. J CLIN EPIDEMIOL 2019, 108:26-33.

25. Shea BJ, Reeves BC, Wells G, Thuku M, Hamel C, Moran J, Moher D, Tugwell

For peer review only

P, Welch V, Kristjansson E et al: AMSTAR 2: a critical appraisal tool for systematic reviews that include randomised or non-randomised studies of healthcare interventions, or both. BMJ 2017, 358:j4008.

Figure caption

Fig 1. PRISMA Flowchart of the study selection process

PRISMA = Preferred Reporting Items for Systematic Reviews and Meta-Analyses, OVCFs = osteoporotic vertebral compression fractures, PVP = percutaneous vertebroplasty, PKP = percutaneous kyphoplasty, SRs/MAs = systematic reviews and meta-analysis

Tables

Table 1. Search strategy for PubMed/MEDLINE®.

#1	"kyphoplasty"[mh] OR "balloon vertebroplasty"[all] OR "vertebroplasty, balloon"[all]
#2	"vertebroplasty"[mh]
#3	#1 AND #2
#4	(((((((percutaneous vertebroplast*[Title/Abstract]) OR pereutancous vertebroplast*[Title/Abstract]) OR vertebroplast*[Title/Abstract]) OR cementoplast*[Title/Abstract]) OR sacroplast*[Title/Abstract]) OR balloon kyphoplast*[Title/Abstract]) OR kyphoplast*[Title/Abstract])
#5	#3 OR #4
#6	"spinal fractures"[mh] OR "fracture, spinal"[all] OR "fractures, spinal"[all] OR "spinal fracture"[all]
#7	"fractures, compression"[mh] OR "compression fracture"[all] OR "fracture, compression"[all] OR "compression fractures"[all]
#8	#6 AND #7
#9	(spinal compression fracture[Title/Abstract]) OR vertebral compression fracture[Title/Abstract]
#10	#8 OR #9
#11	"meta-analysis"[pt] OR "systematic review"[pt] OR "meta-analysis as topic"[mh] OR "systematic reviews as topic"[mh] OR cochrane*[all] OR evidence-based review*[all] OR health technology assessment*[all] OR meta analy*[all] OR meta-review*[all] OR meta-synthes*[all] OR research overview*[all] OR systematic overview*[all] OR systematic review*[all] OR metaanaly*[all] OR meta-analy*[all] OR metanaly*[all]
#12	#5 AND #10 AND #11

For peer review only - http://bmjopen.bmj.com/site/about/guidelines.xhtml

For peer review only

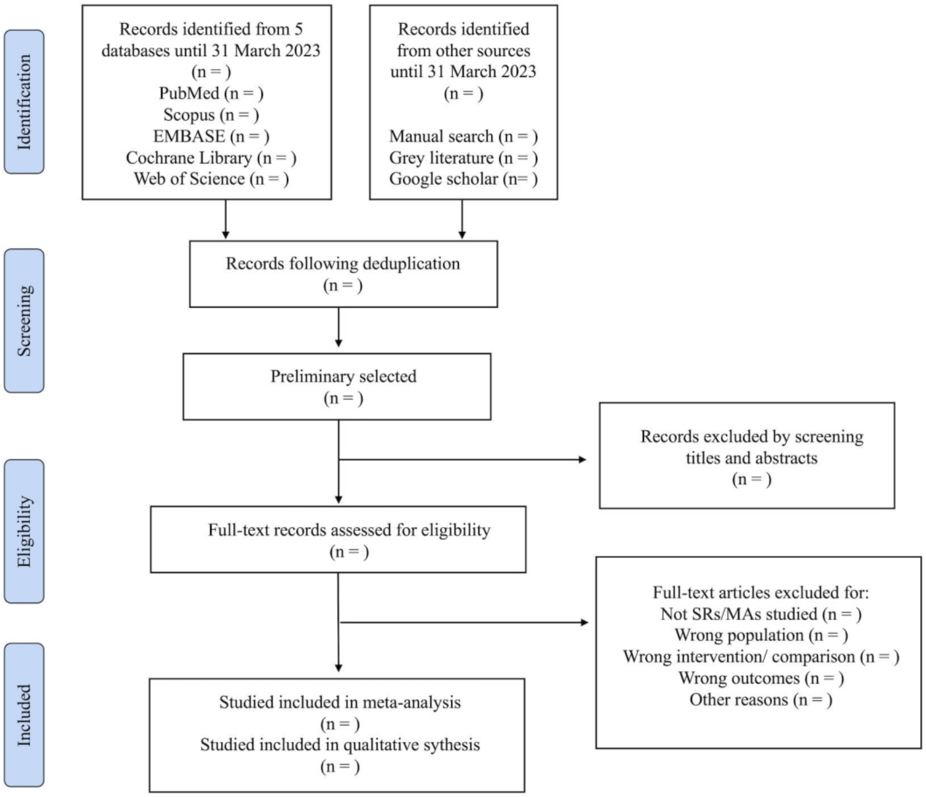

Fig 1. PRISMA Flowchart of study selection process
PRISMA = Preferred Reporting Items for Systematic Reviews and Meta-Analyses, OVCFs = osteoporotic vertebral compression fractures, PVP = percutaneous vertebroplasty, PKP = percutaneous kyphoplasty, SRs/MAs = systematic reviews and meta-analysis

300x261mm (150 x 150 DPI)

PRISMA-P (Preferred Reporting Items for Systematic review and Meta-Analysis Protocols) 2015 checklist: recommended items to address in a systematic review protocol*

Section and topic	Item No	Checklist item	Reported on Page #
**ADMINISTRATIVE INFORMATION**			
Title:			
Identification	1a	Identify the report as a protocol of a systematic review	P.2
Update	1b	If the protocol is for an update of a previous systematic review, identify as such	NA
Registration	2	If registered, provide the name of the registry (such as PROSPERO) and registration number	P.2
Authors:			
Contact	3a	Provide name, institutional affiliation, e-mail address of all protocol authors; provide physical mailing address of corresponding author	P.1
Contributions	3b	Describe contributions of protocol authors and identify the guarantor of the review	P.9
Amendments	4	If the protocol represents an amendment of a previously completed or published protocol, identify as such and list changes; otherwise, state plan for documenting important protocol amendments	NA
Support:			
Sources	5a	Indicate sources of financial or other support for the review	P.9
Sponsor	5b	Provide name for the review funder and/or sponsor	P.9
Role of sponsor or funder	5c	Describe roles of funder(s), sponsor(s), and/or institution(s), if any, in developing the protocol	P.9
**INTRODUCTION**			
Rationale	6	Describe the rationale for the review in the context of what is already known	P.4
Objectives	7	Provide an explicit statement of the question(s) the review will address with reference to participants, interventions, comparators, and outcomes (PICO)	P.5
**METHODS**			
Eligibility criteria	8	Specify the study characteristics (such as PICO, study design, setting, time frame) and report characteristics (such as years considered, language, publication status) to be used as criteria for eligibility for the review	P.5 and 6
Information sources	9	Describe all intended information sources (such as electronic databases, contact with study authors, trial registers or other grey literature sources) with planned dates of coverage	P.5
Search strategy	10	Present draft of search strategy to be used for at least one electronic database, including planned limits, such that it could be repeated	P.5 Figure1

For peer review only

Study records:			
Data management	11a	Describe the mechanism(s) that will be used to manage records and data throughout the review	P.7
Selection process	11b	State the process that will be used for selecting studies (such as two independent reviewers) through each phase of the review (that is, screening, eligibility and inclusion in meta-analysis)	P.7
Data collection process	11c	Describe planned method of extracting data from reports (such as piloting forms, done independently, in duplicate), any processes for obtaining and confirming data from investigators	P.7
Data items	12	List and define all variables for which data will be sought (such as PICO items, funding sources), any pre-planned data assumptions and simplifications	P.7
Outcomes and prioritization	13	List and define all outcomes for which data will be sought, including prioritization of main and additional outcomes, with rationale	P.6
Risk of bias in individual studies	14	Describe anticipated methods for assessing risk of bias of individual studies, including whether this will be done at the outcome or study level, or both; state how this information will be used in data synthesis	P.8
Data synthesis	15a	Describe criteria under which study data will be quantitatively synthesised	P.8
	15b	If data are appropriate for quantitative synthesis, describe planned summary measures, methods of handling data and methods of combining data from studies, including any planned exploration of consistency (such as I^2, Kendall's τ)	P.8
	15c	Describe any proposed additional analyses (such as sensitivity or subgroup analyses, meta-regression)	P.8
	15d	If quantitative synthesis is not appropriate, describe the type of summary planned	P.8
Meta-bias(es)	16	Specify any planned assessment of meta-bias(es) (such as publication bias across studies, selective reporting within studies)	P.8
Confidence in cumulative evidence	17	Describe how the strength of the body of evidence will be assessed (such as GRADE)	P.8

*** It is strongly recommended that this checklist be read in conjunction with the PRISMA-P Explanation and Elaboration (cite when available) for important clarification on the items. Amendments to a review protocol should be tracked and dated.** **The copyright for PRISMA-P (including checklist) is held by the PRISMA-P Group and is distributed under a Creative Commons Attribution Licence 4.0.**

From: Shamseer L, Moher D, Clarke M, Ghersi D, Liberati A, Petticrew M, Shekelle P, Stewart L, PRISMA-P Group. Preferred reporting items for systematic review and meta-analysis protocols (PRISMA-P) 2015: elaboration and explanation. BMJ. 2015 Jan 2;349(jan02 1):g7647.

For peer review only

AMSTAR 2: a critical appraisal tool for systematic reviews that include randomised or non-randomised studies of healthcare interventions, or both

1. Did the research questions and inclusion criteria for the review include the components of PICO?

For Yes:	Optional (recommended)	
☐ Population<br>☐ Intervention<br>☐ Comparator group<br>☐ Outcome	☐ Timeframe for follow-up	☐ Yes<br>☐ No

2. Did the report of the review contain an explicit statement that the review methods were established prior to the conduct of the review and did the report justify any significant deviations from the protocol?

For Partial Yes:	For Yes:	
The authors state that they had a written protocol or guide that included ALL the following:<br>☐ review question(s)<br>☐ a search strategy<br>☐ inclusion/exclusion criteria<br>☐ a risk of bias assessment	As for partial yes, plus the protocol should be registered and should also have specified:<br>☐ a meta-analysis/synthesis plan, if appropriate, *and*<br>☐ a plan for investigating causes of heterogeneity<br>☐ justification for any deviations from the protocol	☐ Yes<br>☐ Partial Yes<br>☐ No

3. Did the review authors explain their selection of the study designs for inclusion in the review?

For Yes, the review should satisfy ONE of the following:	
☐ *Explanation for* including only RCTs<br>☐ OR *Explanation for* including only NRSI<br>☐ OR *Explanation for* including both RCTs and NRSI	☐ Yes<br>☐ No

4. Did the review authors use a comprehensive literature search strategy?

For Partial Yes (all the following):	For Yes, should also have (all the following):	
☐ searched at least 2 databases (relevant to research question)<br>☐ provided key word and/or search strategy<br>☐ justified publication restrictions (e.g. language)	☐ searched the reference lists / bibliographies of included studies<br>☐ searched trial/study registries<br>☐ included/consulted content experts in the field<br>☐ where relevant, searched for grey literature<br>☐ conducted search within 24 months of completion of the review	☐ Yes<br>☐ Partial Yes<br>☐ No

5. Did the review authors perform study selection in duplicate?

For Yes, either ONE of the following:	
☐ at least two reviewers independently agreed on selection of eligible studies and achieved consensus on which studies to include<br>☐ OR two reviewers selected a sample of eligible studies and achieved good agreement (at least 80 percent), with the remainder selected by one reviewer.	☐ Yes<br>☐ No

For peer review only

AMSTAR 2: a critical appraisal tool for systematic reviews that include randomised or non-randomised studies of healthcare interventions, or both

6. Did the review authors perform data extraction in duplicate?

For Yes, either ONE of the following:

- ☐ at least two reviewers achieved consensus on which data to extract from included studies
- ☐ OR two reviewers extracted data from a sample of eligible studies and achieved good agreement (at least 80 percent), with the remainder extracted by one reviewer.

☐ Yes
☐ No

7. Did the review authors provide a list of excluded studies and justify the exclusions?

For Partial Yes:

- ☐ provided a list of all potentially relevant studies that were read in full-text form but excluded from the review

For Yes, must also have:

- ☐ Justified the exclusion from the review of each potentially relevant study

☐ Yes
☐ Partial Yes
☐ No

8. Did the review authors describe the included studies in adequate detail?

For Partial Yes (ALL the following):

- ☐ described populations
- ☐ described interventions
- ☐ described comparators
- ☐ described outcomes
- ☐ described research designs

For Yes, should also have ALL the following:

- ☐ described population in detail
- ☐ described intervention in detail (including doses where relevant)
- ☐ described comparator in detail (including doses where relevant)
- ☐ described study's setting
- ☐ timeframe for follow-up

☐ Yes
☐ Partial Yes
☐ No

9. Did the review authors use a satisfactory technique for assessing the risk of bias (RoB) in individual studies that were included in the review?

RCTs

For Partial Yes, must have assessed RoB from

- ☐ unconcealed allocation, *and*
- ☐ lack of blinding of patients and assessors when assessing outcomes (unnecessary for objective outcomes such as all-cause mortality)

For Yes, must also have assessed RoB from:

- ☐ allocation sequence that was not truly random, *and*
- ☐ selection of the reported result from among multiple measurements or analyses of a specified outcome

☐ Yes
☐ Partial Yes
☐ No
☐ Includes only NRSI

NRSI

For Partial Yes, must have assessed RoB:

- ☐ from confounding, *and*
- ☐ from selection bias

For Yes, must also have assessed RoB:

- ☐ methods used to ascertain exposures and outcomes, *and*
- ☐ selection of the reported result from among multiple measurements or analyses of a specified outcome

☐ Yes
☐ Partial Yes
☐ No
☐ Includes only RCTs

10. Did the review authors report on the sources of funding for the studies included in the review?

For Yes

- ☐ Must have reported on the sources of funding for individual studies included in the review. Note: Reporting that the reviewers looked for this information but it was not reported by study authors also qualifies

☐ Yes
☐ No

For peer review only

AMSTAR 2: a critical appraisal tool for systematic reviews that include randomised or non-randomised studies of healthcare interventions, or both

11. If meta-analysis was performed did the review authors use appropriate methods for statistical combination of results?

For Yes:	
**RCTs**<br>For Yes:<br>☐ The authors justified combining the data in a meta-analysis<br>☐ AND they used an appropriate weighted technique to combine study results and adjusted for heterogeneity if present.<br>☐ AND investigated the causes of any heterogeneity	☐ Yes<br>☐ No<br>☐ No meta-analysis conducted
**For NRSI**<br>For Yes:<br>☐ The authors justified combining the data in a meta-analysis<br>☐ AND they used an appropriate weighted technique to combine study results, adjusting for heterogeneity if present<br>☐ AND they statistically combined effect estimates from NRSI that were adjusted for confounding, rather than combining raw data, or justified combining raw data when adjusted effect estimates were not available<br>☐ AND they reported separate summary estimates for RCTs and NRSI separately when both were included in the review	☐ Yes<br>☐ No<br>☐ No meta-analysis conducted

12. If meta-analysis was performed, did the review authors assess the potential impact of RoB in individual studies on the results of the meta-analysis or other evidence synthesis?

For Yes:	
☐ included only low risk of bias RCTs<br>☐ OR, if the pooled estimate was based on RCTs and/or NRSI at variable RoB, the authors performed analyses to investigate possible impact of RoB on summary estimates of effect.	☐ Yes<br>☐ No<br>☐ No meta-analysis conducted

13. Did the review authors account for RoB in individual studies when interpreting/ discussing the results of the review?

For Yes:	
☐ included only low risk of bias RCTs<br>☐ OR, if RCTs with moderate or high RoB, or NRSI were included the review provided a discussion of the likely impact of RoB on the results	☐ Yes<br>☐ No

14. Did the review authors provide a satisfactory explanation for, and discussion of, any heterogeneity observed in the results of the review?

For Yes:	
☐ There was no significant heterogeneity in the results<br>☐ OR if heterogeneity was present the authors performed an investigation of sources of any heterogeneity in the results and discussed the impact of this on the results of the review	☐ Yes<br>☐ No

15. If they performed quantitative synthesis did the review authors carry out an adequate investigation of publication bias (small study bias) and discuss its likely impact on the results of the review?

For Yes:	
☐ performed graphical or statistical tests for publication bias and discussed the likelihood and magnitude of impact of publication bias	☐ Yes<br>☐ No<br>☐ No meta-analysis conducted

For peer review only

AMSTAR 2: a critical appraisal tool for systematic reviews that include randomised or non-randomised studies of healthcare interventions, or both

16. Did the review authors report any potential sources of conflict of interest, including any funding they received for conducting the review?

For Yes:	
□ The authors reported no competing interests OR	□ Yes
□ The authors described their funding sources and how they managed potential conflicts of interest	□ No

To cite this tool: Shea BJ, Reeves BC, Wells G, Thuku M, Hamel C, Moran J, Moher D, Tugwell P, Welch V, Kristjansson E, Henry DA. AMSTAR 2: a critical appraisal tool for systematic reviews that include randomised or non-randomised studies of healthcare interventions, or both. BMJ. 2017 Sep 21;358:j4008.

For peer review only

AMSTAR 2 contains 16 items, and 7 of them are critical domains (items 2, 4, 7, 9, 11, 13, 15). For more details refer to Table.

Items	Content
2	protocol registered before the beginning of the review
4	adequacy of the literature search
7	justification for excluding individual studies
9	risk of bias (ROB) from individual studies included in the review
11	appropriateness of meta-analytical methods
13	consideration of ROB when interpreting the results of the review
15	assessment of presence and likely impact of publication bias

For peer review only

Percutaneous Vertebroplasty vs Percutaneous Kyphoplasty for Osteoporotic Vertebral Compression Fractures: An Umbrella Review Protocol of Systematic Reviews and Meta-Analyses

Journal:	*BMJ Open*
Manuscript ID	bmjopen-2023-075225.R1
Article Type:	Protocol
Date Submitted by the Author:	10-Dec-2023
Complete List of Authors:	Gao, Qingyang; Sichuan University West China Hospital, Department of Plastic and Burn Surgery<br>Li, Qiujiang; Sichuan University West China Hospital, Department of Orthopedics Surgery, Orthopedic Research Institute<br>Liang, Wang; West China Hospital of Sichuan University, Department of Orthopedics, Orthopedic Research Institute<br>Cen, Ying; Sichuan University West China Hospital, Department of Plastic and Burn Surgery<br>Yang, Huiliang; Sichuan University West China Hospital, Department of Orthopedics, Orthopedic Research Institute,
**Primary Subject Heading**:	Surgery
Secondary Subject Heading:	Surgery
Keywords:	Aging, ORTHOPAEDIC & TRAUMA SURGERY, Bone diseases < ORTHOPAEDIC & TRAUMA SURGERY, Spine < ORTHOPAEDIC & TRAUMA SURGERY

Percutaneous Vertebroplasty vs Percutaneous Kyphoplasty for Osteoporotic Vertebral Compression Fractures: An Umbrella Review Protocol of Systematic Reviews and Meta-Analyses

Qingyang Gao[a,†], Qiujiang Li[b,†], Liang Wang [b,†], Ying Cen[a], Huiliang Yang[b,*]

Abstract

Introduction: Several systematic reviews and meta-analyses have confirmed that percutaneous vertebroplasty and percutaneous kyphoplasty showed safety and beneficial efficacy in patients with osteoporotic vertebral compression fractures. Whereas, there is wide variation among results, which are not conducive to the evaluation and use of clinicians. This study will investigate the efficacy and safety of percutaneous vertebroplasty and percutaneous kyphoplasty for the treatment of osteoporotic vertebral compression fractures, aiming to provide a more reliable evidence base for clinical practice in treating osteoporotic vertebral compression fractures.

Methods and analysis: We will retrieve the relevant articles using the five databases(PubMed, Scopus, EMBASE, Cochrane Library and Web of Science) from inception to March 2023 for systematic review and meta-analysis comparing the overall safety and efficacy of percutaneous vertebroplasty and percutaneous kyphoplasty in patients with osteoporotic vertebral compression fractures. Three reviewers will screen citation titles, abstracts, and evaluate the full text of each relevant citation based on prespecified eligibility criteria. Any discrepancies in decisions between reviewers will be resolved through discussion. We will assess the methodological quality of the included studies according to A MeaSurement Tool to Assess systematic Reviews 2 checklist.

Results: The results of this study will be published in a peer-reviewed journal.

Ethics and dissemination: This umbrella review will inform clinical and policy decisions regarding the benefits and harms of percutaneous vertebroplasty versus percutaneous kyphoplasty for osteoporotic vertebral compression fractures. Neither primary data nor individual patient information will be collected, thus ethics approval is not required. Findings will be reported through a peer-reviewed publication, conference presentations, and the popular press.

Protocol registration number: PROSPERO CRD42021268141 (https://www.crd.york.ac.uk/PROSPERO/#joinuppage)

For peer review only - http://bmjopen.bmj.com/site/about/guidelines.xhtml

Strengths and limitations of this study

1.This will be a comprehensive review assessing the reliability of current evidence on percutaneous vertebroplasty in the management of osteoporotic vertebral compression fractures compared with percutaneous kyphoplasty.

2.This protocol was designed following Joanna Briggs Institute's guidelines for umbrella reviews and reported following the Preferred Reporting Items for Systematic Reviews and Meta-Analyses statement.

3.The results will affect clinical and policy decisions regarding the benefits and harms of percutaneous vertebroplasty for treating osteoporotic vertebral compression fractures.

4.The conclusion of this study might be challenged because of the poor quality and heterogeneity of the reviewed studies.

Abbreviations: OVCFs = osteoporotic vertebral compression fractures, PVP = percutaneous vertebroplasty, PKP = percutaneous kyphoplasty, PMMA = polymethylmethacrylate, RCT = randomized clinical trial, SRs/MAs = systematic reviews and meta-analysis, VAS = visual analog scale, ODI = Oswestry Disability Index, SF-36 = Short Form-36, CI = confidence intervals

Keywords: osteoporotic vertebral compression fractures, umbrella review, percutaneous vertebroplasty, percutaneous kyphoplasty

1. Introduction

As the society ages, osteoporotic vertebral compression fractures (OVCFs) caused by osteoporosis have become a major worldwide health issue[1,2]. OVCFs often occur as a result of low-impact injuries and patients may not have a clear history of trauma. They primarily present with persistent back pain and local vertebral kyphosis, which significantly affects the survival rates and living standards of the elderly population [3–6].

Non-surgical therapies for OVCFs include bed rest and drug therapy[7,8]. However, these methods often provide inadequate pain relief, leaving patients with limitations in their daily activities. Moreover, complications such as pressure sores, pneumonia, deep vein thrombosis, and urinary tract infections may arise during the treatment. Surgical interventions are typically reserved for more severe fractures[9].

For peer review only - http://bmjopen.bmj.com/site/about/guidelines.xhtml

Currently, percutaneous vertebroplasty (PVP) and percutaneous kyphoplasty (PKP) have been widely applied in OVCFs treatment. Both techniques offer immediate pain relief and help stabilize the fractured vertebral body [10–14]. Specifically, PVP involves restoring the vertebral volume by injecting polymethylmethacrylate (PMMA) bone cement through a minimally invasive wound directly into the affected area[15]. PKP represents an advancement over PVP. By placing a balloon into the involved vertebral body before the injection, PKP creates a regularly shaped cavity to control the distribution of the bone cement[15]. Consequently, some researchers believe that PKP should carry a lower risk of bone cement leakage and provide more stable reinforcement than PVP[16–18].

Nonetheless, the debate persists regarding which method, PKP or PVP, is superior for treating OVCFs . In a meta-analysis of six RCT studies involving 1077 patients, Zhu et al. Zhu et al. [19] performed a meta-analysis of six randomized clinical trial (RCT) studies involving a total of 1077 patients and finally found that both PKP and PVP could improve function and relieve pain, but PKP had lower cement leaking rates. By contrast, a meta-analysis of 16 studies aiming to evaluate the efficacy and safety of PKP/PVP for the treatment of OVCFs found that PKP could better reduce bone cement leakage, restore vertebral height restoration and correct vertebral angulation in comparison to PVP [20]. The same conclusion is drawn by Chang et al. [21].

Systematic reviews and meta-analyses (SRs/MAs) are important research methods in evidence-based medicine and the best sources of clinical evidence[22] to create clinical guidelines and develop recommendations for medical practice. Although there are multiple published SRs/MAs studies on PKP/PVP for OVCFs, they produced varying and sometimes conflicting results, which could impact clinical decision-making.

To provide a comprehensive overview of these SRs/MAs studies, an umbrella review (or Systematic Review of Systematic Reviews) is needed. As another important part of evidence-based medicine, an umbrella review assesses the methodological and report quality of included SRs/MAs and provides the quality of evidence. This will be the first study to summarize systematic reviews and meta-analyses on clinical outcomes of PVP and PKP for the treatment of OVCFs. an umbrella review presented in our protocol will identify, collect, evaluate, and integrate the existing knowledge of PKP/PVP for OVCFs to determine the more beneficial surgical treatments between PKP and PVP for patients with OVCFs and guide optimal treatment strategies. The results of this study will provide evidence for clinicians and further guide researchers to embark on relevant studies.

2. Methods and analysis

2.1 Study registration

The study protocol was registered on the International Prospective Register of Systematic Reviews (PROSPERO) (https://www.crd.york.ac.uk/prospero) with a

For peer review only - http://bmjopen.bmj.com/site/about/guidelines.xhtml

unique ID of CRD42021268141. It was designed using the methodology guidelines for umbrella reviews provided by the Joanna Briggs Institute (JBI) [23]. Meanwhile, this protocol will follow the guidance of the Preferred Reporting Items for Systematic Reviews and Meta-analysis Protocols (PRISMA-P) [24] **(see online supplementary appendix 1 for the checklist).**

The research questions of this umbrella review were:

1) What is the efficacy (Primary outcomes) of PCP (Intervention) compared to PVP (Comparison) in OVCFs patients (Population)?

2) What is the safety (Secondary outcomes) of PCP (Intervention) compared to PVP (Comparison) in OVCFs patients (Population)?

3) To identify the pre-, and peri-operative factors (Intervention and Comparison) that affect the functional improvements and radiographic improvements (Outcome) in OVCFs patients (Population).

2.2 Patient and public involvement

No patients or the public will be involved in this study.

2.3 Search strategy

An electronic search will be conducted among various databases, including PubMed 、 Scopus 、 EMBASE 、 Cochrane Library and Web of Science with the Medical Subject Headings (MeSH) and keywords "percutaneous vertebroplasty", "percutaneous kyphoplasty", " balloon kyphoplasty", "osteoporosis vertebral compression fractures", "systematic reviews", "meta-analysis". The terms will be put into combinations with Boolean search syntax operators to retrieve the maximum number of articles.

As a supplement, the "related article" function from PubMed will be used to further identify potential articles that were eligible for inclusion in the review. In addition, grey literature and Google Scholar will also be searched. To include as many relevant studies as possible, we will consult with content experts. A draft search strategy for PubMed is provided in **Table 1.**

2.4 Eligibility criteria

2.4.1 Type of studies

We will include both systematic reviews and meta-analysis studies comparing different clinical outcomes following PKP/PVP.

The studies should report the outcome data separately for the PKP and PVP groups.

For peer review only - http://bmjopen.bmj.com/site/about/guidelines.xhtml

2.4.2 Publication time

The date range will be set from database inception to March 2023.

2.4.3 Language of literature

The language of publication is restricted to English.

2.5 Inclusion criteria

We set the inclusion criteria for this umbrella review according to the population, intervention, comparison, outcome (PICO) principle [25].

2.5.1 Population:

Patients with vertebral compression fractures (VCFs) due to osteoporosis will be included. We impose a lower age limit >= 60 years to identify the elderly. Expect that, there was no restriction on gender, race or healthy status.

2.5.2 Intervention:

Interventions include PVP with curative intent

2.5.3 Comparison:

PKP with curative intent is considered as comparison

The volume of injected cement, fluoroscopy times, intraoperative blood loss and operation time were recorded for both intervention and comparison groups.

2.5.4 Outcomes

Primary outcomes include:

1) Functional outcomes: the visual analog scale (VAS) score, Oswestry Disability Index (ODI), Short Form-36 (SF-36);

2) Radiographic outcomes included Cobb angle and anterior vertebral body height.

Secondary outcomes include:

1) Bone cement leakage;

2) New vertebral compression fractures after the treatment

All outcomes will be assessed based on the definitions applied in the selected meta-analysis. Studies will not be included or excluded on the basis of reported outcomes.

For peer review only - http://bmjopen.bmj.com/site/about/guidelines.xhtml

2.6 Data collection

2.6.1 Study selection

Firstly, literature not meeting the eligibility criteria will be excluded through the screening of titles and abstracts. Subsequently, the full text of preliminarily selected articles will be independently reviewed by two reviewers (QY G and QJ L) to confirm the inclusion and exclusion criteria for enrolled items. Using a data extraction form, two additional reviewers (L W and Y C) will extract data, with subsequent cross-checking by the initial reviewers (QY G and QJ L). If there is a disagreement in the checked results, the arbitration will be resolved through discussion with a third reviewer(HL Y). The flowchart of the literature selection process is shown in **Figure 1.**

2.6.2 Data extraction

Two reviewers (L W and Y C) will extract data independently based on a tool developed by the Joanna Briggs Institute (JBI) Data Extraction form. When data extraction fails to reach an agreement, the arbitration will be resolved through discussion with a third reviewer (HL Y). If data were depicted in graphs or incomplete, we will try to contact authors to obtain original data by email.

Extracted data include:

1) basic information of the literature: first author, publication year range, country;

2) information of the study: study design included, sample size, search strategy， quality evaluation method， original research involved and interest disclosure;

3) population: gender, age, race, pain and deformity before treatment;

4) intervention/comparison: treatment, level of the vertebra, the volume of injected cement, fluoroscopy times, intraoperative blood loss and operation time;

5) outcomes: primary outcome and secondary outcome as described above, time-point to obtain the outcomes;

6) conclusion.

For meta-analysis, we will extract summary effect sizes (random effect size and/or fixed effect size, 95% confidence intervals (CI)) and significance levels. When accessible, information on between-study heterogeneity (Cochrane Q statistic or I^2) and small-study effects will also be gathered.

2.7 Data synthesis and statistical analysis

Firstly, tables and graphs will be used to delineate the characteristics of all eligible studies and provide a concise summary of their findings, guided by the Cochrane

For peer review only - http://bmjopen.bmj.com/site/about/guidelines.xhtml

Handbook [26]. Then, for meta-analysis, further statistical analysis will be conducted. Utilizing both fixed and random effects models, the pooled effect size, 95% CI, p-value, and of the selected meta-analyses will be calculated. Significance levels will be categorized into three thresholds ($p \leqslant 0.05$, $p \leqslant 10^{-3}$, and $p \leqslant 10^{-6}$). Given the potential overlap of individual studies within each meta-analysis, the outcomes will be interpreted with consideration for duplication. Heterogeneity among the included studies will be presented as Cochran's Q statistics and the I^2 index. Egger regression asymmetry test will be used to show the presence of the small study effects. The methodological quality of the meta-analysis will be evaluated using the A Measurement Tool to Assess Systematic Reviews 2 (AMSTAR 2) tool [27].

The relationship and findings between studies will be explored through a narrative synthesis. In the concluding phase of the integrative review, we will articulate and summarize the key findings and conclusions of the studies. Subgroup analysis, taking into account the influence of variables such as sex, age, and outcomes, will be also conducted. The statistical analysis will be performed by using STATA (version 17).

2.7.1 Identifying the overlap of the literature

Given the inevitable duplication of original research in our selected review, we will employ the method outlined by Wegner et. Al[28] to visualize the duplication. In brief, a table will be created with the names of systematic reviews displayed as column headers, and the individual studies from each review listed as row entries. Then, a formula was used to compute the corrected covered area (CCA)[29]:

$$CCA = \frac{N - r}{r(c - r)}$$

Here, N represents the total number of included original publications in the reviews, r denotes the number of single studies of each review (rows), and c signifies the number of systematic reviews (columns).

For those CCA scores >11%, our approach will involve including the most recently published and highest quality review, while excluding other overlapping literature that is homogeneous. Conversely, literature with a CCA score < 5% will prompt a meticulous examination of the search strategy and research scope. CCA scores falling between 5% to 11% will be regarded as indicative of a moderate level of overlap.

2.7.2 Identifying possible publication bias

Reporting bias will be assessed through a funnel plot. To mitigate the potential impact of subjective visual observation, Begg's and Egger's tests will be conducted to evaluate the asymmetry of the funnel plot, as a supplementary approach.

2.7.3 Methodological quality evaluation

Two reviewers independently assess the methodological quality of systematic

For peer review only - http://bmjopen.bmj.com/site/about/guidelines.xhtml

reviews based on AMSTAR 2 [27,29,30]. When quality assessment results fail to reach an agreement, arbitration will be sought through discussion with a third reviewer. The AMSTAR 2 is a reliable and valid critical appraisal tool designed for systematic reviews, applied to both randomized and non-randomized studies of healthcare interventions **(see online supplementary appendix 2 for the checklist) .** AMSTAR 2 comprises 16 items, with 7 identified as critical domains (items 2, 4, 7, 9, 11, 13, 15) [30] . Further details can be found in **online supplementary appendix 3**. The results of evidence quality for systematic reviews will be categorized into four levels according to AMSTAR 2: critically low, low, medium and high.

2.7.4 Grading the evidence

According to the previous studies [31–33], the evidence for outcomes in each meta-analysis was classified as follows:

Convincing (class I): p-value of the random-effect model $< 10^{-6}$, case number > 1000, low heterogeneity between the studies, non-significant bias or small study effect, robustness to unmeasured confounding, significant result ($p < 0.05$) for the largest study, 95% prediction interval not including the null value, and $I^2 < 50\%$;

Highly suggestive (class II): p-value of the random-effect model $< 10^{-6}$, case number > 1000 (or more than 20,000 participants for continuous outcomes), and significant result ($p < 0.05$) for the largest study;

Suggestive (class III): p-value of the random-effect model $< 10^{-3}$, and case number > 1000;

Weak (class IV): evidence was assigned to the remaining significant association with a p-value of the random-effect model < 0.05;

Non-Significant (Class V): p-value for the random effect model > 0.05.

3. Ethics and dissemination

There is no ethical approval or consent required for this systematic review study. The results of our study will be published in an international peer-reviewed journal and disseminated to the public through publications, conferences, and meetings.

4. Authors' information

Qingyang Gao[a,†], Qiujiang Li[b,†], Liang Wang[b,†], Ying Cen[a], Huiliang Yang[b,*]

a. Department of Plastic and Burn Surgery, West China Hospital, Sichuan University, Chengdu, 610041, P.R. China

For peer review only - http://bmjopen.bmj.com/site/about/guidelines.xhtml

b. Department of Orthopedics, Orthopedic Research Institute, West China Hospital, Sichuan University, Chengdu, 610041, P.R. China

* Correspondence:

Huiliang Yang, MD, Department of Orthopedics, Orthopedic Research Institute, West China Hospital, Sichuan University, 610041, P.R. China; Email: huiliang_yang@wchscu.cn

┼ Qingyang Gao, Qiujiang Li and Liang Wang contributed equally to this manuscript

5. Authors' contributions

All authors made substantive intellectual contributions in this study to qualify as authors. QY G and QJ L developed the research question. L W and QJ L registered the protocol and wrote the first draft of the manuscript. QY G and HL Y developed the search strategy and created the data extraction form. Y C and HL Y provided critical review and feedback at each stage of the process. All authors were involved in writing the manuscript. All authors read and approved the final manuscript.

6. Funding statement

This study was funded by The Science and Technology Project of the Health Planning Committee of Sichuan (No. 21PJ036) and the National Natural Science Foundation of China (No. 82102521).

7. Competing interest statement

None declared.

8. Patient consent for publication

Not applicable.

9. References:

For peer review only - http://bmjopen.bmj.com/site/about/guidelines.xhtml

1 Li H-M, Zhang R-J, Gao H, *et al.* New vertebral fractures after osteoporotic vertebral compression fracture between balloon kyphoplasty and nonsurgical treatment PRISMA. *Medicine*. 2018;97:e12666.

2 Jin Y-Z, Lee JH, Xu B, *et al.* Effect of medications on prevention of secondary osteoporotic vertebral compression fracture, non-vertebral fracture, and discontinuation due to adverse events: a meta-analysis of randomized controlled trials. *BMC Musculoskelet Disord*. 2019;20:399.

3 Yang S, Chen C, Wang H, *et al.* A systematic review of unilateral versus bilateral percutaneous vertebroplasty/percutaneous kyphoplasty for osteoporotic vertebral compression fractures. *Acta Orthopaedica et Traumatologica Turcica*. 2017;51:290–7.

4 Bae I-S, Kim JM, Cheong JH, *et al.* Association between cerebral atrophy and osteoporotic vertebral compression fractures. *PLoS ONE*. 2019;14:e0224439.

5 Wu X-G, Zhang D-Y, Zhu B-Q, *et al.* Efficacy of zoledronic acid with percutaneous kyphoplasty/vertebroplasty in the treatment of osteoporotic vertebral compression fractures: a systematic review and meta-analysis. *European Review for Medical and Pharmacological Sciences*. 2020;24:12358–67.

6 Zhu R-S, Kan S-L, Ning G-Z, *et al.* Which is the best treatment of osteoporotic vertebral compression fractures: balloon kyphoplasty, percutaneous vertebroplasty, or non-surgical treatment? A Bayesian network meta-analysis. *Osteoporos Int*. 2019;30:287–98.

7 Long Y, Yi W, Yang D. Advances in Vertebral Augmentation Systems for Osteoporotic Vertebral Compression Fractures. *Pain Res Manag*. 2020;2020:3947368.

8 Pan M, Ge J, Li Q, *et al.* Percutaneous vertebral augmentation in special Genant IV osteoporotic vertebral compression fractures. *Journal of Orthopaedic Translation*. 2020;20:94–9.

9 Klazen CA, Lohle PN, De Vries J, *et al.* Vertebroplasty versus conservative treatment in acute osteoporotic vertebral compression fractures (Vertos II): an open-label randomised trial. *The Lancet*. 2010;376:1085–92.

10 Zuo X-H, Zhu X-P, Bao H-G, *et al.* Network meta-analysis of percutaneous vertebroplasty, percutaneous kyphoplasty, nerve block, and conservative treatment for nonsurgery options of acute/subacute and chronic osteoporotic vertebral compression fractures (OVCFs) in short-term and long-term effects. *Medicine*. 2018;97:e11544.

11 Dai C, Liang G, Zhang Y, *et al.* Risk factors of vertebral re-fracture after PVP or PKP for osteoporotic vertebral compression fractures, especially in Eastern Asia:

For peer review only

a systematic review and meta-analysis. *J Orthop Surg Res*. 2022;17:161.

12 Zhang H, Xu C, Zhang T, *et al.* Does Percutaneous Vertebroplasty or Balloon Kyphoplasty for Osteoporotic Vertebral Compression Fractures Increase the Incidence of New Vertebral Fractures? A Meta-Analysis. *Pain Physician*. 2017;20:E13–28.

13 Xiao H, Yang J, Feng X, *et al.* Comparing complications of vertebroplasty and kyphoplasty for treating osteoporotic vertebral compression fractures: a meta-analysis of the randomized and non-randomized controlled studies. *Eur J Orthop Surg Traumatol*. 2015;25 Suppl 1:S77-85.

14 Wang H, Sribastav SS, Ye F, *et al.* Comparison of Percutaneous Vertebroplasty and Balloon Kyphoplasty for the Treatment of Single Level Vertebral Compression Fractures: A Meta-analysis of the Literature. *Pain Physician*. 2015;18:209–22.

15 Peh WCG, Munk PL, Rashid F, *et al.* Percutaneous Vertebral Augmentation: Vertebroplasty, Kyphoplasty and Skyphoplasty. *Radiologic Clinics of North America*. 2008;46:611–35.

16 Gan M, Zou J, Song D, *et al.* Is balloon kyphoplasty better than percutaneous vertebroplasty for osteoporotic vertebral biconcave-shaped fractures? *Acta Radiol*. 2014;55:985–91.

17 Wang B, Zhao C-P, Song L-X, *et al.* Balloon kyphoplasty versus percutaneous vertebroplasty for osteoporotic vertebral compression fracture: a meta-analysis and systematic review. *J Orthop Surg Res*. 2018;13:264.

18 Shi-Ming G, Wen-Juan L, Yun-Mei H, *et al.* Percutaneous vertebroplasty and percutaneous balloon kyphoplasty for osteoporotic vertebral compression fracture: A metaanalysis. *IJOO*. 2015;49:377–87.

19 Zhu Y, Cheng J, Yin J, *et al.* Therapeutic effect of kyphoplasty and balloon vertebroplasty on osteoporotic vertebral compression fracture: A systematic review and meta-analysis of randomized controlled trials. *Medicine*. 2019;98:e17810.

20 Ma X-L, Xing D, Ma J-X, *et al.* Balloon kyphoplasty versus percutaneous vertebroplasty in treating osteoporotic vertebral compression fracture: grading the evidence through a systematic review and meta-analysis. *Eur Spine J*. 2012;21:1844–59.

21 Chang X, Lv Y-F, Chen B, *et al.* Vertebroplasty versus kyphoplasty in osteoporotic vertebral compression fracture: a meta-analysis of prospective comparative studies. *Int Orthop*. 2015;39:491–500.

22 Shrestha BM. Systematic Reviews and Meta-analysis: Principles and Practice.

For peer review only - http://bmjopen.bmj.com/site/about/guidelines.xhtml

JNMA J Nepal Med Assoc. 2019;57:1–2.

23 Munn Z. Software to support the systematic review process: the Joanna Briggs Institute System for the Unified Management, Assessment and Review of Information (JBI-SUMARI). *JBI Database of Systematic Reviews and Implementation Reports*. 2016;14:1.

24 Moher D, Shamseer L, Clarke M, *et al.* Preferred reporting items for systematic review and meta-analysis protocols (PRISMA-P) 2015 statement. *Syst Rev*. 2015;4:1.

25 Richardson WS, Wilson MC, Nishikawa J, *et al.*, editors. The well-built clinical question: a key to evidence-based decisions. *ACP Journal Club*. 1995;123:A12.

26 Cumpston M, Li T, Page MJ, *et al.* Updated guidance for trusted systematic reviews: a new edition of the Cochrane Handbook for Systematic Reviews of Interventions. *Cochrane Database of Systematic Reviews*. Published Online First: 3 October 2019. doi: 10.1002/14651858.ED000142

27 Kim H-R, Choi C-H, Jo E. A Methodological Quality Assessment of Meta-Analysis Studies in Dance Therapy Using AMSTAR and AMSTAR 2. *Healthcare*. 2020;8:446.

28 Wegner M, Amatriain-Fernández S, Kaulitzky A, *et al.* Systematic Review of Meta-Analyses: Exercise Effects on Depression in Children and Adolescents. *Front Psychiatry*. 2020;11:81.

29 Pieper D, Antoine S-L, Mathes T, *et al.* Systematic review finds overlapping reviews were not mentioned in every other overview. *Journal of Clinical Epidemiology*. 2014;67:368–75.

30 Franco JVA, Meza N. Authors should also report the support for judgment when applying AMSTAR 2. *Journal of Clinical Epidemiology*. 2021;138:240.

31 Kim JY, Son MJ, Son CY, *et al.* Environmental risk factors and biomarkers for autism spectrum disorder: an umbrella review of the evidence. *The Lancet Psychiatry*. 2019;6:590–600.

32 Jenabi E, Salimi Z, Ayubi E, *et al.* The environmental risk factors prior to conception associated with placental abruption: an umbrella review. *Syst Rev*. 2022;11:55.

33 Salehi AM, Jenabi E, Farashi S, *et al.* The environmental risk factors related to uterine leiomyoma: An umbrella review. *Journal of Gynecology Obstetrics and Human Reproduction*. 2023;52:102517.

For peer review only - http://bmjopen.bmj.com/site/about/guidelines.xhtml

Figure Caption:

Fig 1. PRISMA Flowchart of study selection process

PRISMA = Preferred Reporting Items for Systematic Reviews and Meta-Analyses, OVCFs = osteoporotic vertebral compression fractures, PVP = percutaneous vertebroplasty, PKP = percutaneous kyphoplasty, SRs/MAs = systematic reviews and meta-analysis

**Table 1. Search strategy for PubMed/MEDLINE®.**	
#1	"kyphoplasty"[mh] OR "balloon vertebroplasty"[all] OR "vertebroplasty, balloon"[all]
#2	"vertebroplasty"[mh]
#3	#1 AND #2
#4	(((((((percutaneous vertebroplast*[Title/Abstract]) OR pereutancous vertebroplast*[Title/Abstract]) OR vertebroplast*[Title/Abstract]) OR cementoplast*[Title/Abstract]) OR sacroplast*[Title/Abstract]) OR balloon kyphoplast*[Title/Abstract]) OR kyphoplast*[Title/Abstract])
#5	#3 OR #4
#6	"spinal fractures"[mh] OR "fracture, spinal"[all] OR "fractures, spinal"[all] OR "spinal fracture"[all]
#7	"fractures, compression"[mh] OR "compression fracture"[all] OR "fracture, compression"[all] OR "compression fractures"[all]
#8	#6 AND #7
#9	(spinal compression fracture[Title/Abstract]) OR vertebral compression fracture[Title/Abstract]
#10	#8 OR #9
#11	"meta-analysis"[pt] OR "systematic review"[pt] OR "meta-analysis as topic"[mh] OR "systematic reviews as topic"[mh] OR cochrane*[all] OR evidence-based review*[all] OR health technology assessment*[all] OR meta analy*[all] OR meta-review*[all] OR meta-synthes*[all] OR research overview*[all] OR systematic overview*[all] OR systematic review*[all] OR metaanaly*[all] OR meta-analy*[all] OR metanaly*[all]
#12	#5 AND #10 AND #11

Tables:

For peer review only - http://bmjopen.bmj.com/site/about/guidelines.xhtml

For peer review only

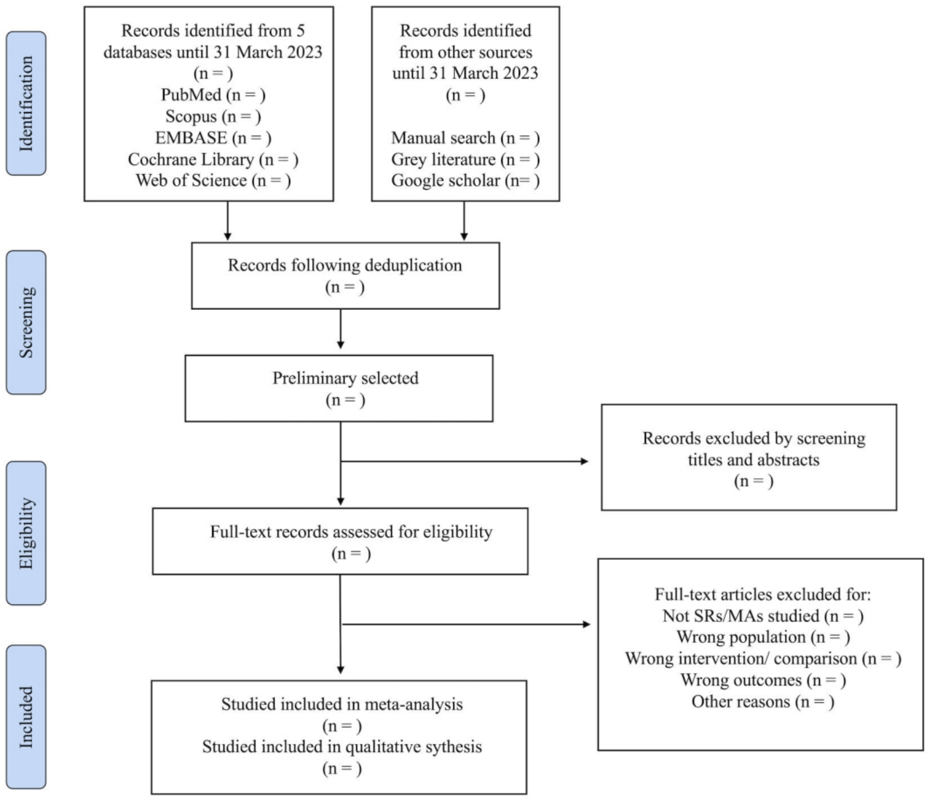

Fig 1. PRISMA Flowchart of study selection process
PRISMA = Preferred Reporting Items for Systematic Reviews and Meta-Analyses, OVCFs = osteoporotic vertebral compression fractures, PVP = percutaneous vertebroplasty, PKP = percutaneous kyphoplasty, SRs/MAs = systematic reviews and meta-analysis

300x261mm (150 x 150 DPI)

PRISMA-P (Preferred Reporting Items for Systematic review and Meta-Analysis Protocols) 2015 checklist: recommended items to address in a systematic review protocol*

Section and topic	Item No	Checklist item	Reported on Page #
**ADMINISTRATIVE INFORMATION**			
Title:			
Identification	1a	Identify the report as a protocol of a systematic review	P.2
Update	1b	If the protocol is for an update of a previous systematic review, identify as such	NA
Registration	2	If registered, provide the name of the registry (such as PROSPERO) and registration number	P.2
Authors:			
Contact	3a	Provide name, institutional affiliation, e-mail address of all protocol authors; provide physical mailing address of corresponding author	P.1
Contributions	3b	Describe contributions of protocol authors and identify the guarantor of the review	P.9
Amendments	4	If the protocol represents an amendment of a previously completed or published protocol, identify as such and list changes; otherwise, state plan for documenting important protocol amendments	NA
Support:			
Sources	5a	Indicate sources of financial or other support for the review	P.9
Sponsor	5b	Provide name for the review funder and/or sponsor	P.9
Role of sponsor or funder	5c	Describe roles of funder(s), sponsor(s), and/or institution(s), if any, in developing the protocol	P.9
**INTRODUCTION**			
Rationale	6	Describe the rationale for the review in the context of what is already known	P.4
Objectives	7	Provide an explicit statement of the question(s) the review will address with reference to participants, interventions, comparators, and outcomes (PICO)	P.5
**METHODS**			
Eligibility criteria	8	Specify the study characteristics (such as PICO, study design, setting, time frame) and report characteristics (such as years considered, language, publication status) to be used as criteria for eligibility for the review	P.5 and 6
Information sources	9	Describe all intended information sources (such as electronic databases, contact with study authors, trial registers or other grey literature sources) with planned dates of coverage	P.5
Search strategy	10	Present draft of search strategy to be used for at least one electronic database, including planned limits, such that it could be repeated	P.5 Figure1

For peer review only

Study records:			
Data management	11a	Describe the mechanism(s) that will be used to manage records and data throughout the review	P.7
Selection process	11b	State the process that will be used for selecting studies (such as two independent reviewers) through each phase of the review (that is, screening, eligibility and inclusion in meta-analysis)	P.7
Data collection process	11c	Describe planned method of extracting data from reports (such as piloting forms, done independently, in duplicate), any processes for obtaining and confirming data from investigators	P.7
Data items	12	List and define all variables for which data will be sought (such as PICO items, funding sources), any pre-planned data assumptions and simplifications	P.7
Outcomes and prioritization	13	List and define all outcomes for which data will be sought, including prioritization of main and additional outcomes, with rationale	P.6
Risk of bias in individual studies	14	Describe anticipated methods for assessing risk of bias of individual studies, including whether this will be done at the outcome or study level, or both; state how this information will be used in data synthesis	P.8
Data synthesis	15a	Describe criteria under which study data will be quantitatively synthesised	P.8
	15b	If data are appropriate for quantitative synthesis, describe planned summary measures, methods of handling data and methods of combining data from studies, including any planned exploration of consistency (such as I^2, Kendall's τ)	P.8
	15c	Describe any proposed additional analyses (such as sensitivity or subgroup analyses, meta-regression)	P.8
	15d	If quantitative synthesis is not appropriate, describe the type of summary planned	P.8
Meta-bias(es)	16	Specify any planned assessment of meta-bias(es) (such as publication bias across studies, selective reporting within studies)	P.8
Confidence in cumulative evidence	17	Describe how the strength of the body of evidence will be assessed (such as GRADE)	P.8

*** It is strongly recommended that this checklist be read in conjunction with the PRISMA-P Explanation and Elaboration (cite when available) for important clarification on the items. Amendments to a review protocol should be tracked and dated. The copyright for PRISMA-P (including checklist) is held by the PRISMA-P Group and is distributed under a Creative Commons Attribution Licence 4.0.**

From: Shamseer L, Moher D, Clarke M, Ghersi D, Liberati A, Petticrew M, Shekelle P, Stewart L, PRISMA-P Group. Preferred reporting items for systematic review and meta-analysis protocols (PRISMA-P) 2015: elaboration and explanation. BMJ. 2015 Jan 2;349(jan02 1):g7647.

For peer review only

AMSTAR 2: a critical appraisal tool for systematic reviews that include randomised or non-randomised studies of healthcare interventions, or both

1. Did the research questions and inclusion criteria for the review include the components of PICO?

For Yes:	Optional (recommended)	
☐ Population<br>☐ Intervention<br>☐ Comparator group<br>☐ Outcome	☐ Timeframe for follow-up	☐ Yes<br>☐ No

2. Did the report of the review contain an explicit statement that the review methods were established prior to the conduct of the review and did the report justify any significant deviations from the protocol?

For Partial Yes:	For Yes:	
The authors state that they had a written protocol or guide that included ALL the following:<br>☐ review question(s)<br>☐ a search strategy<br>☐ inclusion/exclusion criteria<br>☐ a risk of bias assessment	As for partial yes, plus the protocol should be registered and should also have specified:<br>☐ a meta-analysis/synthesis plan, if appropriate, *and*<br>☐ a plan for investigating causes of heterogeneity<br>☐ justification for any deviations from the protocol	☐ Yes<br>☐ Partial Yes<br>☐ No

3. Did the review authors explain their selection of the study designs for inclusion in the review?

For Yes, the review should satisfy ONE of the following:	
☐ *Explanation for* including only RCTs<br>☐ OR *Explanation for* including only NRSI<br>☐ OR *Explanation for* including both RCTs and NRSI	☐ Yes<br>☐ No

4. Did the review authors use a comprehensive literature search strategy?

For Partial Yes (all the following):	For Yes, should also have (all the following):	
☐ searched at least 2 databases (relevant to research question)<br>☐ provided key word and/or search strategy<br>☐ justified publication restrictions (e.g. language)	☐ searched the reference lists / bibliographies of included studies<br>☐ searched trial/study registries<br>☐ included/consulted content experts in the field<br>☐ where relevant, searched for grey literature<br>☐ conducted search within 24 months of completion of the review	☐ Yes<br>☐ Partial Yes<br>☐ No

5. Did the review authors perform study selection in duplicate?

For Yes, either ONE of the following:	
☐ at least two reviewers independently agreed on selection of eligible studies and achieved consensus on which studies to include<br>☐ OR two reviewers selected a sample of eligible studies and achieved good agreement (at least 80 percent), with the remainder selected by one reviewer.	☐ Yes<br>☐ No

For peer review only

AMSTAR 2: a critical appraisal tool for systematic reviews that include randomised or non-randomised studies of healthcare interventions, or both

6. Did the review authors perform data extraction in duplicate?

For Yes, either ONE of the following:	
☐ at least two reviewers achieved consensus on which data to extract from included studies<br>☐ OR two reviewers extracted data from a sample of eligible studies <u>and</u> achieved good agreement (at least 80 percent), with the remainder extracted by one reviewer.	☐ Yes<br>☐ No

7. Did the review authors provide a list of excluded studies and justify the exclusions?

For Partial Yes:	For Yes, must also have:	
☐ provided a list of all potentially relevant studies that were read in full-text form but excluded from the review	☐ Justified the exclusion from the review of each potentially relevant study	☐ Yes<br>☐ Partial Yes<br>☐ No

8. Did the review authors describe the included studies in adequate detail?

For Partial Yes (ALL the following):	For Yes, should also have ALL the following:	
☐ described populations<br>☐ described interventions<br>☐ described comparators<br>☐ described outcomes<br>☐ described research designs	☐ described population in detail<br>☐ described intervention in detail (including doses where relevant)<br>☐ described comparator in detail (including doses where relevant)<br>☐ described study's setting<br>☐ timeframe for follow-up	☐ Yes<br>☐ Partial Yes<br>☐ No

9. Did the review authors use a satisfactory technique for assessing the risk of bias (RoB) in individual studies that were included in the review?

**RCTs**<br>For Partial Yes, must have assessed RoB from	For Yes, must also have assessed RoB from:	
☐ unconcealed allocation, *and*<br>☐ lack of blinding of patients and assessors when assessing outcomes (unnecessary for objective outcomes such as all-cause mortality)	☐ allocation sequence that was not truly random, *and*<br>☐ selection of the reported result from among multiple measurements or analyses of a specified outcome	☐ Yes<br>☐ Partial Yes<br>☐ No<br>☐ Includes only NRSI
**NRSI**<br>For Partial Yes, must have assessed RoB:	For Yes, must also have assessed RoB:	
☐ from confounding, *and*<br>☐ from selection bias	☐ methods used to ascertain exposures and outcomes, *and*<br>☐ selection of the reported result from among multiple measurements or analyses of a specified outcome	☐ Yes<br>☐ Partial Yes<br>☐ No<br>☐ Includes only RCTs

10. Did the review authors report on the sources of funding for the studies included in the review?

For Yes	
☐ Must have reported on the sources of funding for individual studies included in the review. Note: Reporting that the reviewers looked for this information but it was not reported by study authors also qualifies	☐ Yes<br>☐ No

For peer review only

AMSTAR 2: a critical appraisal tool for systematic reviews that include randomised or non-randomised studies of healthcare interventions, or both

11. If meta-analysis was performed did the review authors use appropriate methods for statistical combination of results?

RCTs
For Yes:
- ☐ The authors justified combining the data in a meta-analysis
  - ☐ AND they used an appropriate weighted technique to combine study results and adjusted for heterogeneity if present.
  - ☐ AND investigated the causes of any heterogeneity

☐ Yes
☐ No
☐ No meta-analysis conducted

For NRSI
For Yes:
- ☐ The authors justified combining the data in a meta-analysis
  - ☐ AND they used an appropriate weighted technique to combine study results, adjusting for heterogeneity if present
  - ☐ AND they statistically combined effect estimates from NRSI that were adjusted for confounding, rather than combining raw data, or justified combining raw data when adjusted effect estimates were not available
  - ☐ AND they reported separate summary estimates for RCTs and NRSI separately when both were included in the review

☐ Yes
☐ No
☐ No meta-analysis conducted

12. If meta-analysis was performed, did the review authors assess the potential impact of RoB in individual studies on the results of the meta-analysis or other evidence synthesis?

For Yes:
- ☐ included only low risk of bias RCTs
- ☐ OR, if the pooled estimate was based on RCTs and/or NRSI at variable RoB, the authors performed analyses to investigate possible impact of RoB on summary estimates of effect.

☐ Yes
☐ No
☐ No meta-analysis conducted

13. Did the review authors account for RoB in individual studies when interpreting/ discussing the results of the review?

For Yes:
- ☐ included only low risk of bias RCTs
- ☐ OR, if RCTs with moderate or high RoB, or NRSI were included the review provided a discussion of the likely impact of RoB on the results

☐ Yes
☐ No

14. Did the review authors provide a satisfactory explanation for, and discussion of, any heterogeneity observed in the results of the review?

For Yes:
- ☐ There was no significant heterogeneity in the results
- ☐ OR if heterogeneity was present the authors performed an investigation of sources of any heterogeneity in the results and discussed the impact of this on the results of the review

☐ Yes
☐ No

15. If they performed quantitative synthesis did the review authors carry out an adequate investigation of publication bias (small study bias) and discuss its likely impact on the results of the review?

For Yes:
- ☐ performed graphical or statistical tests for publication bias and discussed the likelihood and magnitude of impact of publication bias

☐ Yes
☐ No
☐ No meta-analysis conducted

For peer review only

AMSTAR 2: a critical appraisal tool for systematic reviews that include randomised or non-randomised studies of healthcare interventions, or both

16. Did the review authors report any potential sources of conflict of interest, including any funding they received for conducting the review?

For Yes:	
☐ The authors reported no competing interests OR	☐ Yes
☐ The authors described their funding sources and how they managed potential conflicts of interest	☐ No

To cite this tool: Shea BJ, Reeves BC, Wells G, Thuku M, Hamel C, Moran J, Moher D, Tugwell P, Welch V, Kristjansson E, Henry DA. AMSTAR 2: a critical appraisal tool for systematic reviews that include randomised or non-randomised studies of healthcare interventions, or both. BMJ. 2017 Sep 21;358:j4008.

For peer review only

AMSTAR 2 contains 16 items, and 7 of them are critical domains (items 2, 4, 7, 9, 11, 13, 15). For more details refer to Table.

Items	Content
2	protocol registered before the beginning of the review
4	adequacy of the literature search
7	justification for excluding individual studies
9	risk of bias (ROB) from individual studies included in the review
11	appropriateness of meta-analytical methods
13	consideration of ROB when interpreting the results of the review
15	assessment of presence and likely impact of publication bias

For peer review only

BMJ Open

Percutaneous Vertebroplasty vs Percutaneous Kyphoplasty for Osteoporotic Vertebral Compression Fractures: An Umbrella Review Protocol of Systematic Reviews and Meta-Analyses

Journal:	*BMJ Open*
Manuscript ID	bmjopen-2023-075225.R2
Article Type:	Protocol
Date Submitted by the Author:	30-Dec-2023
Complete List of Authors:	Gao, Qingyang; Sichuan University West China Hospital, Department of Plastic and Burn Surgery<br>Li, Qiujiang; Sichuan University West China Hospital, Department of Orthopedic Surgery, Orthopedic Research Institute<br>Liang, Wang; West China Hospital of Sichuan University, Department of Orthopedic Surgery, Orthopedic Research Institute<br>Cen, Ying; Sichuan University West China Hospital, Department of Plastic and Burn Surgery<br>Yang, Huiliang; Sichuan University West China Hospital, Department of Orthopedic Surgery, Orthopedic Research Institute
**Primary Subject Heading**:	Surgery
Secondary Subject Heading:	Surgery
Keywords:	Aging, ORTHOPAEDIC & TRAUMA SURGERY, Bone diseases < ORTHOPAEDIC & TRAUMA SURGERY, Spine < ORTHOPAEDIC & TRAUMA SURGERY

SCHOLARONE™ Manuscripts

Percutaneous Vertebroplasty vs Percutaneous Kyphoplasty for Osteoporotic Vertebral Compression Fractures: An Umbrella Review Protocol of Systematic Reviews and Meta-Analyses

Qingyang Gao[a,+], Qiujiang Li[b,+], Liang Wang [b,+], Ying Cen[a], Huiliang Yang[b,*]

a. Department of Plastic and Burn Surgery, West China Hospital, Sichuan University, Chengdu, 610041, P.R. China

b. Department of Orthopedic Surgery, Orthopedic Research Institute, West China Hospital, Sichuan University, Chengdu, 610041, P.R. China

*** Correspondence:**

Huiliang Yang, MD, Department of Orthopedics, Orthopedic Research Institute, West China Hospital, Sichuan University, 610041, P.R. China; Email: huiliang_yang@wchscu.cn

┼ Qingyang Gao, Qiujiang Li and Liang Wang contributed equally to this manuscript.

Keywords: Spinal Fractures, Osteoporotic Fractures, Umbrella Review, Percutaneous Vertebroplasty, Percutaneous Kyphoplasty

Word count: 3982

For peer review only

Percutaneous Vertebroplasty vs Percutaneous Kyphoplasty for Osteoporotic Vertebral Compression Fractures: An Umbrella Review Protocol of Systematic Reviews and Meta-Analyses

Abstract

Introduction: Several systematic reviews and meta-analyses have confirmed that percutaneous vertebroplasty and percutaneous kyphoplasty showed safety and beneficial efficacy in patients with osteoporotic vertebral compression fractures. Whereas, there is wide variation among results, which are not conducive to the evaluation and use of clinicians. This study will investigate the efficacy and safety of percutaneous vertebroplasty and percutaneous kyphoplasty for the treatment of osteoporotic vertebral compression fractures, aiming to provide a more reliable evidence base for clinical practice in treating osteoporotic vertebral compression fractures.

Methods and analysis: We will retrieve the relevant articles using the five databases(PubMed, Scopus, EMBASE, Cochrane Library and Web of Science) from inception to March 2023 for systematic review and meta-analysis comparing the overall safety and efficacy of percutaneous vertebroplasty and percutaneous kyphoplasty in patients with osteoporotic vertebral compression fractures. Three reviewers will screen citation titles, abstracts, and evaluate the full text of each relevant citation based on prespecified eligibility criteria. Any discrepancies in decisions between reviewers will be resolved through discussion. We will assess the methodological quality of the included studies according to A MeaSurement Tool to Assess systematic Reviews 2 checklist.

Ethics and dissemination: This umbrella review will inform clinical and policy decisions regarding the benefits and harms of percutaneous vertebroplasty versus percutaneous kyphoplasty for osteoporotic vertebral compression fractures. Neither primary data nor individual patient information will be collected, thus ethics approval is not required. Findings will be reported through a peer-reviewed publication, conference presentations, and the popular press.

Protocol registration number: PROSPERO CRD42021268141 (https://www.crd.york.ac.uk/PROSPERO/#joinuppage)

For peer review only - http://bmjopen.bmj.com/site/about/guidelines.xhtml

Strengths and limitations of this study

1. This research will conduct a comprehensive review of both meta-analyses and systematic reviews to assess the reliability of current evidence on percutaneous vertebroplasty in the management of osteoporotic vertebral compression fractures compared with percutaneous kyphoplasty.

2. Screening, data collection, and quality assessment were independently conducted by two individuals during the study.

3. Each included review will undergo quality assessment and evidence grading as part of the study protocol.

4. Only literature published in English will be included.

Abbreviations: OVCFs = osteoporotic vertebral compression fractures, PVP = percutaneous vertebroplasty, PKP = percutaneous kyphoplasty, PMMA = polymethylmethacrylate, RCT = randomized clinical trial, SRs/MAs = systematic reviews and meta-analysis, VAS = visual analog scale, ODI = Oswestry Disability Index, SF-36 = Short Form-36, CI = confidence intervals

Keywords: Spinal Fractures, Osteoporotic Fractures, Umbrella Review, Percutaneous Vertebroplasty, Percutaneous Kyphoplasty

1. Introduction

As the society ages, osteoporotic vertebral compression fractures (OVCFs) caused by osteoporosis have become a major worldwide health issue[1,2]. OVCFs often occur as a result of low-impact injuries and patients may not have a clear history of trauma. They primarily present with persistent back pain and local vertebral kyphosis, which significantly affects the survival rates and living standards of the elderly population [3–6].

Non-surgical therapies for OVCFs include bed rest and drug therapy[7,8]. However, these methods often provide inadequate pain relief, leaving patients with limitations in their daily activities. Moreover, complications such as pressure sores,

For peer review only - http://bmjopen.bmj.com/site/about/guidelines.xhtml

pneumonia, deep vein thrombosis, and urinary tract infections may arise during the treatment. Surgical interventions are typically reserved for more severe fractures[9]. Currently, percutaneous vertebroplasty (PVP) and percutaneous kyphoplasty (PKP) have been widely applied in OVCFs treatment. Both techniques offer immediate pain relief and help stabilize the fractured vertebral body [10–14]. Specifically, PVP involves restoring the vertebral volume by injecting polymethylmethacrylate (PMMA) bone cement through a minimally invasive wound directly into the affected area[15]. PKP represents an advancement over PVP. By placing a balloon into the involved vertebral body before the injection, PKP creates a regularly shaped cavity to control the distribution of the bone cement[15]. Consequently, some researchers believe that PKP should carry a lower risk of bone cement leakage and provide more stable reinforcement than PVP[16–18].

Nonetheless, the debate persists regarding which method, PKP or PVP, is superior for treating OVCFs . In a meta-analysis of six RCT studies involving 1077 patients, Zhu et al. Zhu et al. [19] performed a meta-analysis of six randomized clinical trial (RCT) studies involving a total of 1077 patients and finally found that both PKP and PVP could improve function and relieve pain, but PKP had lower cement leaking rates. By contrast, a meta-analysis of 16 studies aiming to evaluate the efficacy and safety of PKP/PVP for the treatment of OVCFs found that PKP could better reduce bone cement leakage, restore vertebral height restoration and correct vertebral angulation in comparison to PVP [20]. The same conclusion is drawn by Chang et al. [21].

Systematic reviews and meta-analyses (SRs/MAs) are important research methods in evidence-based medicine and the best sources of clinical evidence[22] to create clinical guidelines and develop recommendations for medical practice. Although there are multiple published SRs/MAs studies on PKP/PVP for OVCFs, they produced varying and sometimes conflicting results, which could impact clinical decision-making.

To provide a comprehensive overview of these SRs/MAs studies, an umbrella review (or Systematic Review of Systematic Reviews) is needed. As another important part of evidence-based medicine, an umbrella review assesses the methodological and report quality of included SRs/MAs and provides the quality of evidence. This will be the first study to summarize systematic reviews and meta-analyses on clinical outcomes of PVP and PKP for the treatment of OVCFs. an umbrella review presented in our protocol will identify, collect, evaluate, and integrate the existing knowledge of PKP/PVP for OVCFs to determine the more beneficial surgical treatments between PKP and PVP for patients with OVCFs and guide optimal treatment strategies. The results of this study will provide evidence for clinicians and further guide researchers to embark on relevant studies.

2. Methods and analysis

2.1 Study registration

For peer review only - http://bmjopen.bmj.com/site/about/guidelines.xhtml

The study protocol was registered on the International Prospective Register of Systematic Reviews (PROSPERO) (https://www.crd.york.ac.uk/prospero) with a unique ID of CRD42021268141. It was designed using the methodology guidelines for umbrella reviews provided by the Joanna Briggs Institute (JBI) [23]. Meanwhile, this protocol will follow the guidance of the Preferred Reporting Items for Systematic Reviews and Meta-analysis Protocols (PRISMA-P) [24] **(see online supplementary appendix 1 for the checklist).**

The research questions of this umbrella review were:

1) What is the efficacy (Primary outcomes) of PCP (Intervention) compared to PVP (Comparison) in OVCFs patients (Population)?

2) What is the safety (Secondary outcomes) of PCP (Intervention) compared to PVP (Comparison) in OVCFs patients (Population)?

3) To identify the pre-, and peri-operative factors (Intervention and Comparison) that affect the functional improvements and radiographic improvements (Outcome) in OVCFs patients (Population).

2.2 Patient and public involvement

No patients or the public will be involved in this study.

2.3 Search strategy

An electronic search will be conducted among various databases, including PubMed、Scopus、EMBASE、Cochrane Library and Web of Science with the Medical Subject Headings (MeSH) and keywords “percutaneous vertebroplasty”, “percutaneous kyphoplasty”, “ balloon kyphoplasty”, “osteoporosis vertebral compression fractures”, “systematic reviews”, “meta-analysis”. The terms will be put into combinations with Boolean search syntax operators to retrieve the maximum number of articles.

As a supplement, the “related article” function from PubMed will be used to further identify potential articles that were eligible for inclusion in the review. In addition, grey literature and Google Scholar will also be searched. To include as many relevant studies as possible, we will consult with content experts. A draft search strategy for PubMed is provided in **Table 1.**

2.4 Eligibility criteria

2.4.1 Type of studies

We will include both systematic reviews and meta-analysis studies comparing different clinical outcomes following PKP/PVP.

For peer review only - http://bmjopen.bmj.com/site/about/guidelines.xhtml

The studies should report the outcome data separately for the PKP and PVP groups.

2.4.2 Publication time

The date range will be set from database inception to March 2023.

2.4.3 Language of literature

The language of publication is restricted to English.

2.5 Inclusion criteria

We set the inclusion criteria for this umbrella review according to the population, intervention, comparison, outcome (PICO) principle [25].

2.5.1 Population:

Patients with vertebral compression fractures (VCFs) due to osteoporosis will be included. We impose a lower age limit >= 60 years to identify the elderly. Expect that, there was no restriction on gender, race or healthy status.

2.5.2 Intervention:

Interventions include PVP with curative intent

2.5.3 Comparison:

PKP with curative intent is considered as comparison

The volume of injected cement, fluoroscopy times, intraoperative blood loss and operation time were recorded for both intervention and comparison groups.

2.5.4 Outcomes

Primary outcomes include:

1) Functional outcomes: the visual analog scale (VAS) score, Oswestry Disability Index (ODI), Short Form-36 (SF-36);

2) Radiographic outcomes included Cobb angle and anterior vertebral body height.

Secondary outcomes include:

1) Bone cement leakage;

2) New vertebral compression fractures after the treatment

All outcomes will be assessed based on the definitions applied in the selected meta-analysis. Studies will not be included or excluded on the basis of reported

For peer review only - http://bmjopen.bmj.com/site/about/guidelines.xhtml

For peer review only

outcomes.

2.6 Data collection

2.6.1 Study selection

Firstly, literature not meeting the eligibility criteria will be excluded through the screening of titles and abstracts. Subsequently, the full text of preliminarily selected articles will be independently reviewed by two reviewers (QY G and QJ L) to confirm the inclusion and exclusion criteria for enrolled items. Using a data extraction form, two additional reviewers (L W and Y C) will extract data, with subsequent cross-checking by the initial reviewers (QY G and QJ L). If there is a disagreement in the checked results, the arbitration will be resolved through discussion with a third reviewer(HL Y). The flowchart of the literature selection process is shown in **Figure 1.**

2.6.2 Data extraction

Two reviewers (L W and Y C) will extract data independently based on a tool developed by the Joanna Briggs Institute (JBI) Data Extraction form. When data extraction fails to reach an agreement, the arbitration will be resolved through discussion with a third reviewer (HL Y). If data were depicted in graphs or incomplete, we will try to contact authors to obtain original data by email.

Extracted data include:

1) basic information of the literature: first author, publication year range, country;

2) information of the study: study design included, sample size, search strategy， quality evaluation method， original research involved and interest disclosure;

3) population: gender, age, race, pain and deformity before treatment;

4) intervention/comparison: treatment, level of the vertebra, the volume of injected cement, fluoroscopy times, intraoperative blood loss and operation time;

5) outcomes: primary outcome and secondary outcome as described above, time-point to obtain the outcomes;

6) conclusion.

For meta-analysis, we will extract summary effect sizes (random effect size and/or fixed effect size, 95% confidence intervals (CI)) and significance levels. When accessible, information on between-study heterogeneity (Cochrane Q statistic or I^2) and small-study effects will also be gathered.

2.7 Data synthesis and statistical analysis

For peer review only - http://bmjopen.bmj.com/site/about/guidelines.xhtml

Firstly, tables and graphs will be used to delineate the characteristics of all eligible studies and provide a concise summary of their findings, guided by the Cochrane Handbook [26]. Then, for meta-analysis, further statistical analysis will be conducted. Utilizing both fixed and random effects models, the pooled effect size, 95% CI, p-value, and of the selected meta-analyses will be calculated. Significance levels will be categorized into three thresholds ($p \leqslant 0.05$, $p \leqslant 10^{-3}$, and $p \leqslant 10^{-6}$). Given the potential overlap of individual studies within each meta-analysis, the outcomes will be interpreted with consideration for duplication. Heterogeneity among the included studies will be presented as Cochran's Q statistics and the I^2 index. Egger regression asymmetry test will be used to show the presence of the small study effects. The methodological quality of the meta-analysis will be evaluated using the A Measurement Tool to Assess Systematic Reviews 2 (AMSTAR 2) tool [27].

The relationship and findings between studies will be explored through a narrative synthesis. In the concluding phase of the integrative review, we will articulate and summarize the key findings and conclusions of the studies. Subgroup analysis, taking into account the influence of variables such as sex, age, and outcomes, will be also conducted. The statistical analysis will be performed by using STATA (version 17).

2.7.1 Identifying the overlap of the literature

Given the inevitable duplication of original research in our selected review, we will employ the method outlined by Wegner et. Al[28] to visualize the duplication. In brief, a table will be created with the names of systematic reviews displayed as column headers, and the individual studies from each review listed as row entries. Then, a formula was used to compute the corrected covered area (CCA)[29]:

$$CCA = \frac{N - r}{r(c - r)}$$

Here, N represents the total number of included original publications in the reviews, r denotes the number of single studies of each review (rows), and c signifies the number of systematic reviews (columns).

For those CCA scores >11%, our approach will involve including the most recently published and highest quality review, while excluding other overlapping literature that is homogeneous. Conversely, literature with a CCA score < 5% will prompt a meticulous examination of the search strategy and research scope. CCA scores falling between 5% to 11% will be regarded as indicative of a moderate level of overlap.

2.7.2 Identifying possible publication bias

Reporting bias will be assessed through a funnel plot. To mitigate the potential impact of subjective visual observation, Begg's and Egger's tests will be conducted to evaluate the asymmetry of the funnel plot, as a supplementary approach.

For peer review only - http://bmjopen.bmj.com/site/about/guidelines.xhtml

For peer review only

2.7.3 Methodological quality evaluation

Two reviewers independently assess the methodological quality of systematic reviews based on AMSTAR 2 [27,29,30]. When quality assessment results fail to reach an agreement, arbitration will be sought through discussion with a third reviewer. The AMSTAR 2 is a reliable and valid critical appraisal tool designed for systematic reviews, applied to both randomized and non-randomized studies of healthcare interventions **(see online supplementary appendix 2 for the checklist) .** AMSTAR 2 comprises 16 items, with 7 identified as critical domains (items 2, 4, 7, 9, 11, 13, 15) [30] . Further details can be found in **online supplementary appendix 3**. The results of evidence quality for systematic reviews will be categorized into four levels according to AMSTAR 2: critically low, low, medium and high.

2.7.4 Grading the evidence

According to the previous studies [31–33], the evidence for outcomes in each meta-analysis was classified as follows:

Convincing (class I): p-value of the random-effect model $< 10^{-6}$, case number > 1000, low heterogeneity between the studies, non-significant bias or small study effect, robustness to unmeasured confounding, significant result ($p < 0.05$) for the largest study, 95% prediction interval not including the null value, and $I^2 < 50\%$;

Highly suggestive (class II): p-value of the random-effect model $< 10^{-6}$, case number > 1000 (or more than 20,000 participants for continuous outcomes), and significant result ($p < 0.05$) for the largest study;

Suggestive (class III): p-value of the random-effect model $< 10^{-3}$, and case number > 1000;

Weak (class IV): evidence was assigned to the remaining significant association with a p-value of the random-effect model < 0.05;

Non-Significant (Class V): p-value for the random effect model > 0.05.

3. Ethics and dissemination

There is no ethical approval or consent required for this systematic review study. The results of our study will be published in an international peer-reviewed journal and disseminated to the public through publications, conferences, and meetings.

4. Authors' information

Qingyang Gao[a,†], Qiujiang Li[b,†], Liang Wang[b,†], Ying Cen[a], Huiliang Yang[b,*]

For peer review only - http://bmjopen.bmj.com/site/about/guidelines.xhtml

a. Department of Plastic and Burn Surgery, West China Hospital, Sichuan University, Chengdu, 610041, P.R. China

b. Department of Orthopedic Surgery, Orthopedic Research Institute, West China Hospital, Sichuan University, Chengdu, 610041, P.R. China

* Correspondence:

Huiliang Yang, MD, Department of Orthopedics, Orthopedic Research Institute, West China Hospital, Sichuan University, 610041, P.R. China; Email: huiliang_yang@wchscu.cn

┼ Qingyang Gao, Qiujiang Li and Liang Wang contributed equally to this manuscript.

5. Authors' contributions

All authors made substantive intellectual contributions in this study to qualify as authors. QY G and QJ L developed the research question. L W and QJ L registered the protocol and wrote the first draft of the manuscript. QY G and HL Y developed the search strategy and created the data extraction form. Y C and HL Y provided critical review and feedback at each stage of the process. All authors were involved in writing the manuscript. All authors read and approved the final manuscript.

6. Funding statement

This study was funded by The Science and Technology Project of the Health Planning Committee of Sichuan (No. 21PJ036) and the National Natural Science Foundation of China (No. 82102521).

7. Competing interest statement

None declared.

8. Patient consent for publication

Not applicable.

For peer review only

9. References:

1 Li H-M, Zhang R-J, Gao H, *et al.* New vertebral fractures after osteoporotic vertebral compression fracture between balloon kyphoplasty and nonsurgical treatment PRISMA. *Medicine*. 2018;97:e12666.

2 Jin Y-Z, Lee JH, Xu B, *et al.* Effect of medications on prevention of secondary osteoporotic vertebral compression fracture, non-vertebral fracture, and discontinuation due to adverse events: a meta-analysis of randomized controlled trials. *BMC Musculoskelet Disord*. 2019;20:399.

3 Yang S, Chen C, Wang H, *et al.* A systematic review of unilateral versus bilateral percutaneous vertebroplasty/percutaneous kyphoplasty for osteoporotic vertebral compression fractures. *Acta Orthopaedica et Traumatologica Turcica*. 2017;51:290–7.

4 Bae I-S, Kim JM, Cheong JH, *et al.* Association between cerebral atrophy and osteoporotic vertebral compression fractures. *PLoS ONE*. 2019;14:e0224439.

5 Wu X-G, Zhang D-Y, Zhu B-Q, *et al.* Efficacy of zoledronic acid with percutaneous kyphoplasty/vertebroplasty in the treatment of osteoporotic vertebral compression fractures: a systematic review and meta-analysis. *European Review for Medical and Pharmacological Sciences*. 2020;24:12358–67.

6 Zhu R-S, Kan S-L, Ning G-Z, *et al.* Which is the best treatment of osteoporotic vertebral compression fractures: balloon kyphoplasty, percutaneous vertebroplasty, or non-surgical treatment? A Bayesian network meta-analysis. *Osteoporos Int*. 2019;30:287–98.

7 Long Y, Yi W, Yang D. Advances in Vertebral Augmentation Systems for Osteoporotic Vertebral Compression Fractures. *Pain Res Manag*. 2020;2020:3947368.

8 Pan M, Ge J, Li Q, *et al.* Percutaneous vertebral augmentation in special Genant IV osteoporotic vertebral compression fractures. *Journal of Orthopaedic Translation*. 2020;20:94–9.

9 Klazen CA, Lohle PN, De Vries J, *et al.* Vertebroplasty versus conservative treatment in acute osteoporotic vertebral compression fractures (Vertos II): an open-label randomised trial. *The Lancet*. 2010;376:1085–92.

10 Zuo X-H, Zhu X-P, Bao H-G, *et al.* Network meta-analysis of percutaneous vertebroplasty, percutaneous kyphoplasty, nerve block, and conservative treatment for nonsurgery options of acute/subacute and chronic osteoporotic vertebral compression fractures (OVCFs) in short-term and long-term effects. *Medicine*. 2018;97:e11544.

For peer review only - http://bmjopen.bmj.com/site/about/guidelines.xhtml

11 Dai C, Liang G, Zhang Y, *et al.* Risk factors of vertebral re-fracture after PVP or PKP for osteoporotic vertebral compression fractures, especially in Eastern Asia: a systematic review and meta-analysis. *J Orthop Surg Res*. 2022;17:161.

12 Zhang H, Xu C, Zhang T, *et al.* Does Percutaneous Vertebroplasty or Balloon Kyphoplasty for Osteoporotic Vertebral Compression Fractures Increase the Incidence of New Vertebral Fractures? A Meta-Analysis. *Pain Physician*. 2017;20:E13–28.

13 Xiao H, Yang J, Feng X, *et al.* Comparing complications of vertebroplasty and kyphoplasty for treating osteoporotic vertebral compression fractures: a meta-analysis of the randomized and non-randomized controlled studies. *Eur J Orthop Surg Traumatol*. 2015;25 Suppl 1:S77-85.

14 Wang H, Sribastav SS, Ye F, *et al.* Comparison of Percutaneous Vertebroplasty and Balloon Kyphoplasty for the Treatment of Single Level Vertebral Compression Fractures: A Meta-analysis of the Literature. *Pain Physician*. 2015;18:209–22.

15 Peh WCG, Munk PL, Rashid F, *et al.* Percutaneous Vertebral Augmentation: Vertebroplasty, Kyphoplasty and Skyphoplasty. *Radiologic Clinics of North America*. 2008;46:611–35.

16 Gan M, Zou J, Song D, *et al.* Is balloon kyphoplasty better than percutaneous vertebroplasty for osteoporotic vertebral biconcave-shaped fractures? *Acta Radiol*. 2014;55:985–91.

17 Wang B, Zhao C-P, Song L-X, *et al.* Balloon kyphoplasty versus percutaneous vertebroplasty for osteoporotic vertebral compression fracture: a meta-analysis and systematic review. *J Orthop Surg Res*. 2018;13:264.

18 Shi-Ming G, Wen-Juan L, Yun-Mei H, *et al.* Percutaneous vertebroplasty and percutaneous balloon kyphoplasty for osteoporotic vertebral compression fracture: A metaanalysis. *IJOO*. 2015;49:377–87.

19 Zhu Y, Cheng J, Yin J, *et al.* Therapeutic effect of kyphoplasty and balloon vertebroplasty on osteoporotic vertebral compression fracture: A systematic review and meta-analysis of randomized controlled trials. *Medicine*. 2019;98:e17810.

20 Ma X-L, Xing D, Ma J-X, *et al.* Balloon kyphoplasty versus percutaneous vertebroplasty in treating osteoporotic vertebral compression fracture: grading the evidence through a systematic review and meta-analysis. *Eur Spine J*. 2012;21:1844–59.

21 Chang X, Lv Y-F, Chen B, *et al.* Vertebroplasty versus kyphoplasty in osteoporotic vertebral compression fracture: a meta-analysis of prospective comparative studies. *Int Orthop*. 2015;39:491–500.

For peer review only - http://bmjopen.bmj.com/site/about/guidelines.xhtml

22 Shrestha BM. Systematic Reviews and Meta-analysis: Principles and Practice. *JNMA J Nepal Med Assoc*. 2019;57:1–2.

23 Munn Z. Software to support the systematic review process: the Joanna Briggs Institute System for the Unified Management, Assessment and Review of Information (JBI-SUMARI). *JBI Database of Systematic Reviews and Implementation Reports*. 2016;14:1.

24 Moher D, Shamseer L, Clarke M, *et al.* Preferred reporting items for systematic review and meta-analysis protocols (PRISMA-P) 2015 statement. *Syst Rev*. 2015;4:1.

25 Richardson WS, Wilson MC, Nishikawa J, *et al.*, editors. The well-built clinical question: a key to evidence-based decisions. *ACP Journal Club*. 1995;123:A12.

26 Cumpston M, Li T, Page MJ, *et al.* Updated guidance for trusted systematic reviews: a new edition of the Cochrane Handbook for Systematic Reviews of Interventions. *Cochrane Database of Systematic Reviews*. Published Online First: 3 October 2019. doi: 10.1002/14651858.ED000142

27 Kim H-R, Choi C-H, Jo E. A Methodological Quality Assessment of Meta-Analysis Studies in Dance Therapy Using AMSTAR and AMSTAR 2. *Healthcare*. 2020;8:446.

28 Wegner M, Amatriain-Fernández S, Kaulitzky A, *et al.* Systematic Review of Meta-Analyses: Exercise Effects on Depression in Children and Adolescents. *Front Psychiatry*. 2020;11:81.

29 Pieper D, Antoine S-L, Mathes T, *et al.* Systematic review finds overlapping reviews were not mentioned in every other overview. *Journal of Clinical Epidemiology*. 2014;67:368–75.

30 Franco JVA, Meza N. Authors should also report the support for judgment when applying AMSTAR 2. *Journal of Clinical Epidemiology*. 2021;138:240.

31 Kim JY, Son MJ, Son CY, *et al.* Environmental risk factors and biomarkers for autism spectrum disorder: an umbrella review of the evidence. *The Lancet Psychiatry*. 2019;6:590–600.

32 Jenabi E, Salimi Z, Ayubi E, *et al.* The environmental risk factors prior to conception associated with placental abruption: an umbrella review. *Syst Rev*. 2022;11:55.

33 Salehi AM, Jenabi E, Farashi S, *et al.* The environmental risk factors related to uterine leiomyoma: An umbrella review. *Journal of Gynecology Obstetrics and Human Reproduction*. 2023;52:102517.

For peer review only - http://bmjopen.bmj.com/site/about/guidelines.xhtml

Figure Caption:

Fig 1. PRISMA Flowchart of study selection process

PRISMA = Preferred Reporting Items for Systematic Reviews and Meta-Analyses, OVCFs = osteoporotic vertebral compression fractures, PVP = percutaneous vertebroplasty, PKP = percutaneous kyphoplasty, SRs/MAs = systematic reviews and meta-analysis

Table 1. Search strategy for PubMed/MEDLINE®.

#1	“kyphoplasty”[mh] OR “balloon vertebroplasty”[all] OR “vertebroplasty, balloon”[all]
#2	“vertebroplasty”[mh]
#3	#1 AND #2
#4	(((((((percutaneous vertebroplast*[Title/Abstract]) OR pereutancous vertebroplast*[Title/Abstract]) OR vertebroplast*[Title/Abstract]) OR cementoplast*[Title/Abstract]) OR sacroplast*[Title/Abstract]) OR balloon kyphoplast*[Title/Abstract]) OR kyphoplast*[Title/Abstract])
#5	#3 OR #4
#6	“spinal fractures”[mh] OR “fracture, spinal”[all] OR “fractures, spinal”[all] OR “spinal fracture”[all]
#7	“fractures, compression”[mh] OR “compression fracture”[all] OR “fracture, compression”[all] OR “compression fractures”[all]
#8	#6 AND #7
#9	(spinal compression fracture[Title/Abstract]) OR vertebral compression fracture[Title/Abstract]
#10	#8 OR #9
#11	"meta-analysis"[pt] OR "systematic review"[pt] OR "meta-analysis as topic"[mh] OR "systematic reviews as topic"[mh] OR cochrane*[all] OR evidence-based review*[all] OR health technology assessment*[all] OR meta analy*[all] OR meta-review*[all] OR meta-synthes*[all] OR research overview*[all] OR systematic overview*[all] OR systematic review*[all] OR metaanaly*[all] OR meta-analy*[all] OR metanaly*[all]
#12	#5 AND #10 AND #11

Tables:

For peer review only - http://bmjopen.bmj.com/site/about/guidelines.xhtml

1

2

3

For peer review only - http://bmjopen.bmj.com/site/about/guidelines.xhtml

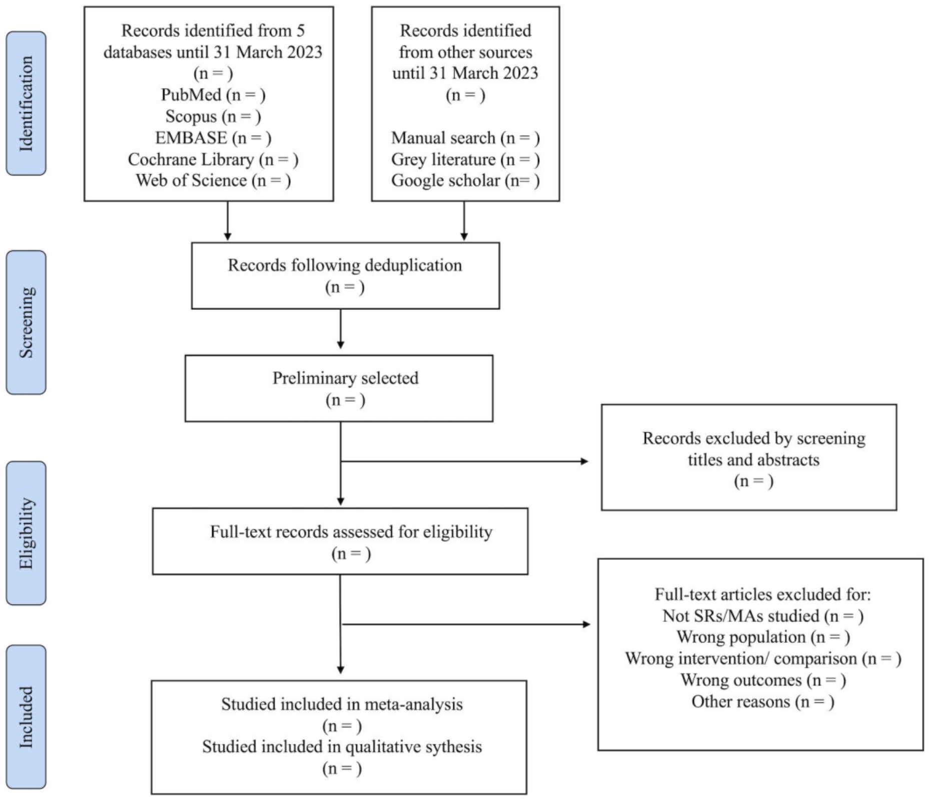

Fig 1. PRISMA Flowchart of study selection process
PRISMA = Preferred Reporting Items for Systematic Reviews and Meta-Analyses, OVCFs = osteoporotic vertebral compression fractures, PVP = percutaneous vertebroplasty, PKP = percutaneous kyphoplasty, SRs/MAs = systematic reviews and meta-analysis

300x261mm (150 x 150 DPI)

PRISMA-P (Preferred Reporting Items for Systematic review and Meta-Analysis Protocols) 2015 checklist: recommended items to address in a systematic review protocol*

Section and topic	Item No	Checklist item	Reported on Page #
**ADMINISTRATIVE INFORMATION**			
Title:			
Identification	1a	Identify the report as a protocol of a systematic review	P.2
Update	1b	If the protocol is for an update of a previous systematic review, identify as such	NA
Registration	2	If registered, provide the name of the registry (such as PROSPERO) and registration number	P.2
Authors:			
Contact	3a	Provide name, institutional affiliation, e-mail address of all protocol authors; provide physical mailing address of corresponding author	P.1
Contributions	3b	Describe contributions of protocol authors and identify the guarantor of the review	P.9
Amendments	4	If the protocol represents an amendment of a previously completed or published protocol, identify as such and list changes; otherwise, state plan for documenting important protocol amendments	NA
Support:			
Sources	5a	Indicate sources of financial or other support for the review	P.9
Sponsor	5b	Provide name for the review funder and/or sponsor	P.9
Role of sponsor or funder	5c	Describe roles of funder(s), sponsor(s), and/or institution(s), if any, in developing the protocol	P.9
**INTRODUCTION**			
Rationale	6	Describe the rationale for the review in the context of what is already known	P.4
Objectives	7	Provide an explicit statement of the question(s) the review will address with reference to participants, interventions, comparators, and outcomes (PICO)	P.5
**METHODS**			
Eligibility criteria	8	Specify the study characteristics (such as PICO, study design, setting, time frame) and report characteristics (such as years considered, language, publication status) to be used as criteria for eligibility for the review	P.5 and 6
Information sources	9	Describe all intended information sources (such as electronic databases, contact with study authors, trial registers or other grey literature sources) with planned dates of coverage	P.5
Search strategy	10	Present draft of search strategy to be used for at least one electronic database, including planned limits, such that it could be repeated	P.5 Figure1

For peer review only

Study records:			
Data management	11a	Describe the mechanism(s) that will be used to manage records and data throughout the review	P.7
Selection process	11b	State the process that will be used for selecting studies (such as two independent reviewers) through each phase of the review (that is, screening, eligibility and inclusion in meta-analysis)	P.7
Data collection process	11c	Describe planned method of extracting data from reports (such as piloting forms, done independently, in duplicate), any processes for obtaining and confirming data from investigators	P.7
Data items	12	List and define all variables for which data will be sought (such as PICO items, funding sources), any pre-planned data assumptions and simplifications	P.7
Outcomes and prioritization	13	List and define all outcomes for which data will be sought, including prioritization of main and additional outcomes, with rationale	P.6
Risk of bias in individual studies	14	Describe anticipated methods for assessing risk of bias of individual studies, including whether this will be done at the outcome or study level, or both; state how this information will be used in data synthesis	P.8
Data synthesis	15a	Describe criteria under which study data will be quantitatively synthesised	P.8
	15b	If data are appropriate for quantitative synthesis, describe planned summary measures, methods of handling data and methods of combining data from studies, including any planned exploration of consistency (such as I^2, Kendall's τ)	P.8
	15c	Describe any proposed additional analyses (such as sensitivity or subgroup analyses, meta-regression)	P.8
	15d	If quantitative synthesis is not appropriate, describe the type of summary planned	P.8
Meta-bias(es)	16	Specify any planned assessment of meta-bias(es) (such as publication bias across studies, selective reporting within studies)	P.8
Confidence in cumulative evidence	17	Describe how the strength of the body of evidence will be assessed (such as GRADE)	P.8

*** It is strongly recommended that this checklist be read in conjunction with the PRISMA-P Explanation and Elaboration (cite when available) for important clarification on the items. Amendments to a review protocol should be tracked and dated.** **The copyright for PRISMA-P (including checklist) is held by the PRISMA-P Group and is distributed under a Creative Commons Attribution Licence 4.0.**

From: Shamseer L, Moher D, Clarke M, Ghersi D, Liberati A, Petticrew M, Shekelle P, Stewart L, PRISMA-P Group. Preferred reporting items for systematic review and meta-analysis protocols (PRISMA-P) 2015: elaboration and explanation. BMJ. 2015 Jan 2;349(jan02 1):g7647.

For peer review only

AMSTAR 2: a critical appraisal tool for systematic reviews that include randomised or non-randomised studies of healthcare interventions, or both

1. Did the research questions and inclusion criteria for the review include the components of PICO?

For Yes:	Optional (recommended)	
☐ Population<br>☐ Intervention<br>☐ Comparator group<br>☐ Outcome	☐ Timeframe for follow-up	☐ Yes<br>☐ No

2. Did the report of the review contain an explicit statement that the review methods were established prior to the conduct of the review and did the report justify any significant deviations from the protocol?

For Partial Yes:	For Yes:	
The authors state that they had a written protocol or guide that included ALL the following:<br>☐ review question(s)<br>☐ a search strategy<br>☐ inclusion/exclusion criteria<br>☐ a risk of bias assessment	As for partial yes, plus the protocol should be registered and should also have specified:<br>☐ a meta-analysis/synthesis plan, if appropriate, *and*<br>☐ a plan for investigating causes of heterogeneity<br>☐ justification for any deviations from the protocol	☐ Yes<br>☐ Partial Yes<br>☐ No

3. Did the review authors explain their selection of the study designs for inclusion in the review?

For Yes, the review should satisfy ONE of the following:	
☐ *Explanation for* including only RCTs<br>☐ OR *Explanation for* including only NRSI<br>☐ OR *Explanation for* including both RCTs and NRSI	☐ Yes<br>☐ No

4. Did the review authors use a comprehensive literature search strategy?

For Partial Yes (all the following):	For Yes, should also have (all the following):	
☐ searched at least 2 databases (relevant to research question)<br>☐ provided key word and/or search strategy<br>☐ justified publication restrictions (e.g. language)	☐ searched the reference lists / bibliographies of included studies<br>☐ searched trial/study registries<br>☐ included/consulted content experts in the field<br>☐ where relevant, searched for grey literature<br>☐ conducted search within 24 months of completion of the review	☐ Yes<br>☐ Partial Yes<br>☐ No

5. Did the review authors perform study selection in duplicate?

For Yes, either ONE of the following:	
☐ at least two reviewers independently agreed on selection of eligible studies and achieved consensus on which studies to include<br>☐ OR two reviewers selected a sample of eligible studies and achieved good agreement (at least 80 percent), with the remainder selected by one reviewer.	☐ Yes<br>☐ No

For peer review only

AMSTAR 2: a critical appraisal tool for systematic reviews that include randomised or non-randomised studies of healthcare interventions, or both

6. Did the review authors perform data extraction in duplicate?

For Yes, either ONE of the following:	
☐ at least two reviewers achieved consensus on which data to extract from included studies<br>☐ OR two reviewers extracted data from a sample of eligible studies <u>and</u> achieved good agreement (at least 80 percent), with the remainder extracted by one reviewer.	☐ Yes<br>☐ No

7. Did the review authors provide a list of excluded studies and justify the exclusions?

For Partial Yes:	For Yes, must also have:	
☐ provided a list of all potentially relevant studies that were read in full-text form but excluded from the review	☐ Justified the exclusion from the review of each potentially relevant study	☐ Yes<br>☐ Partial Yes<br>☐ No

8. Did the review authors describe the included studies in adequate detail?

For Partial Yes (ALL the following):	For Yes, should also have ALL the following:	
☐ described populations<br>☐ described interventions<br>☐ described comparators<br>☐ described outcomes<br>☐ described research designs	☐ described population in detail<br>☐ described intervention in detail (including doses where relevant)<br>☐ described comparator in detail (including doses where relevant)<br>☐ described study's setting<br>☐ timeframe for follow-up	☐ Yes<br>☐ Partial Yes<br>☐ No

9. Did the review authors use a satisfactory technique for assessing the risk of bias (RoB) in individual studies that were included in the review?

**RCTs**<br>For Partial Yes, must have assessed RoB from	For Yes, must also have assessed RoB from:	
☐ unconcealed allocation, *and*<br>☐ lack of blinding of patients and assessors when assessing outcomes (unnecessary for objective outcomes such as all-cause mortality)	☐ allocation sequence that was not truly random, *and*<br>☐ selection of the reported result from among multiple measurements or analyses of a specified outcome	☐ Yes<br>☐ Partial Yes<br>☐ No<br>☐ Includes only NRSI
**NRSI**<br>For Partial Yes, must have assessed RoB:	For Yes, must also have assessed RoB:	
☐ from confounding, *and*<br>☐ from selection bias	☐ methods used to ascertain exposures and outcomes, *and*<br>☐ selection of the reported result from among multiple measurements or analyses of a specified outcome	☐ Yes<br>☐ Partial Yes<br>☐ No<br>☐ Includes only RCTs

10. Did the review authors report on the sources of funding for the studies included in the review?

For Yes	
☐ Must have reported on the sources of funding for individual studies included in the review. Note: Reporting that the reviewers looked for this information but it was not reported by study authors also qualifies	☐ Yes<br>☐ No

For peer review only

AMSTAR 2: a critical appraisal tool for systematic reviews that include randomised or non-randomised studies of healthcare interventions, or both

11. If meta-analysis was performed did the review authors use appropriate methods for statistical combination of results?

RCTs
For Yes:

- ☐ The authors justified combining the data in a meta-analysis
  - ☐ AND they used an appropriate weighted technique to combine study results and adjusted for heterogeneity if present.
  - ☐ AND investigated the causes of any heterogeneity

☐ Yes
☐ No
☐ No meta-analysis conducted

For NRSI
For Yes:

- ☐ The authors justified combining the data in a meta-analysis
  - ☐ AND they used an appropriate weighted technique to combine study results, adjusting for heterogeneity if present
  - ☐ AND they statistically combined effect estimates from NRSI that were adjusted for confounding, rather than combining raw data, or justified combining raw data when adjusted effect estimates were not available
  - ☐ AND they reported separate summary estimates for RCTs and NRSI separately when both were included in the review

☐ Yes
☐ No
☐ No meta-analysis conducted

12. If meta-analysis was performed, did the review authors assess the potential impact of RoB in individual studies on the results of the meta-analysis or other evidence synthesis?

For Yes:

- ☐ included only low risk of bias RCTs
- ☐ OR, if the pooled estimate was based on RCTs and/or NRSI at variable RoB, the authors performed analyses to investigate possible impact of RoB on summary estimates of effect.

☐ Yes
☐ No
☐ No meta-analysis conducted

13. Did the review authors account for RoB in individual studies when interpreting/ discussing the results of the review?

For Yes:

- ☐ included only low risk of bias RCTs
- ☐ OR, if RCTs with moderate or high RoB, or NRSI were included the review provided a discussion of the likely impact of RoB on the results

☐ Yes
☐ No

14. Did the review authors provide a satisfactory explanation for, and discussion of, any heterogeneity observed in the results of the review?

For Yes:

- ☐ There was no significant heterogeneity in the results
- ☐ OR if heterogeneity was present the authors performed an investigation of sources of any heterogeneity in the results and discussed the impact of this on the results of the review

☐ Yes
☐ No

15. If they performed quantitative synthesis did the review authors carry out an adequate investigation of publication bias (small study bias) and discuss its likely impact on the results of the review?

For Yes:

- ☐ performed graphical or statistical tests for publication bias and discussed the likelihood and magnitude of impact of publication bias

☐ Yes
☐ No
☐ No meta-analysis conducted

For peer review only

AMSTAR 2: a critical appraisal tool for systematic reviews that include randomised or non-randomised studies of healthcare interventions, or both

16. Did the review authors report any potential sources of conflict of interest, including any funding they received for conducting the review?

For Yes:	
☐ The authors reported no competing interests OR	☐ Yes
☐ The authors described their funding sources and how they managed potential conflicts of interest	☐ No

To cite this tool: Shea BJ, Reeves BC, Wells G, Thuku M, Hamel C, Moran J, Moher D, Tugwell P, Welch V, Kristjansson E, Henry DA. AMSTAR 2: a critical appraisal tool for systematic reviews that include randomised or non-randomised studies of healthcare interventions, or both. BMJ. 2017 Sep 21;358:j4008.

For peer review only

AMSTAR 2 contains 16 items, and 7 of them are critical domains (items 2, 4, 7, 9, 11, 13, 15). For more details refer to Table.

Items	Content
2	protocol registered before the beginning of the review
4	adequacy of the literature search
7	justification for excluding individual studies
9	risk of bias (ROB) from individual studies included in the review
11	appropriateness of meta-analytical methods
13	consideration of ROB when interpreting the results of the review
15	assessment of presence and likely impact of publication bias

For peer review only
